# Current state of alpaca reproduction: present status and future perspectives of assisted reproductive technologies

**DOI:** 10.3389/fvets.2026.1921209

**Published:** 2026-08-25

**Authors:** Julio Landinez-Aponte, Zhongde Wang

**Affiliations:** Department of Animal, Dairy, and Veterinary Sciences, S.J. and Jessie E. Quinney College of Agriculture and Natural Resources, Utah State University, Logan, UT, United States

**Keywords:** alpaca reproduction, assisted reproductive technologies, follicular wave synchronization, oocyte maturation, *in vitro* fertilization, *in vitro* embryo production, semen processing

## Abstract

The alpaca (*Vicugna pacos*) is a domesticated South American camelid of increasing global relevance, valued for its high-quality fiber, cultural significance, and unique biological traits including heavy-chain-only antibodies. Its reproductive physiology differs fundamentally from that of conventional livestock, encompassing an induced rather than spontaneous ovulatory mechanism in females and an unusually viscous ejaculate in males, differences that constrain the direct application of protocols developed for cattle, sheep, and swine and that have historically limited the pace of genetic improvement in the species. This review provides an integrated account of alpaca reproductive anatomy, follicular and neuroendocrine physiology, seminal plasma biochemistry, and semen cryopreservation, together with the current state of artificial insemination, multiple ovulation and embryo transfer, laparoscopic ovum pick-up, *in vitro* embryo production, and intracytoplasmic sperm injection. By examining female and male reproductive biology within a single mechanistic framework rather than treating each in isolation, this review draws out the regulatory pathways that connect the two systems and identifies the knowledge gaps that a fragmented, single-sex treatment of the subject would otherwise leave unaddressed. Particular attention is given to the biological determinants that most directly limit ART efficiency and to the translational steps required to move current protocols from experimental proof of concept toward standardized, field-applicable practice. Building on prior reviews that have illuminated individual ART modalities and single-sex reproductive biology, this work is intended to support researchers and practitioners advancing reproductive technology in camelids and the broader effort to conserve and genetically improve alpaca populations worldwide.

## 1 Introduction

The alpaca (*Vicugna pacos*) is a domesticated South American camelid (SAC) of considerable scientific and economic significance. Native to the high-altitude Andean plateau, the species has expanded well beyond its geographic origin and is now raised across multiple continents for its high-quality fiber, its cultural relevance to Andean communities, and, increasingly, it’s potential as a model organism for studying unique biological traits (1, 2). Among these traits, the alpaca immune system is particularly noteworthy for harboring a class of heavy-chain-only antibodies; their isolated variable domains (VHH), produced as single-domain fragments, are known as nanobodies, which have attracted strong interest in the fields of diagnostics and biopharmaceutical development (3). In parallel, the reproductive physiology of this species exhibits characteristics markedly distinct from those of conventional livestock, positioning it as a compelling yet technically challenging model for reproductive biotechnology.

Reproductive efficiency in alpacas is frequently limited under both natural and controlled breeding conditions. Reduced conception rates, prolonged postpartum intervals, and a high degree of variability in semen and oocyte quality collectively hinder genetic progress within alpaca populations (4–6). These constraints are exacerbated by the comparatively limited body of research available for this species relative to conventional livestock such as cattle, sheep, and swine, for which robust ART platforms have been established over the last several decades. As a result, reproductive management of alpacas continues to rely predominantly on natural mating, a practice that, while operationally simple, inherently limits the rate of genetic improvement and precludes efficient dissemination of superior germplasm (7).

The biological idiosyncrasies of alpaca reproduction impose substantial constraints on the direct application of ARTs developed for other ruminants. Alpacas are obligate induced ovulators requiring physical or chemical stimulation to trigger ovulation (8, 9), exhibit complex follicular dynamics characterized by prolonged follicular phases and overlapping waves, and lack a clearly defined estrous cycle, complicating the timing of ovulation induction, insemination, and embryo collection (10, 11). On the male side, ejaculates are typically low in volume and highly viscous due to bulbourethral gland secretions, impairing sperm processing and cryopreservation; no universally effective viscosity-reduction method has been established, and many interventions compromise sperm membrane integrity (12, 13). Female reproductive biotechnology faces equally significant challenges, with *in vitro* oocyte maturation and fertilization success rates substantially below those reported for cattle or sheep, and LOPU yielding variable results across laboratories (5, 14–18).

Building on the foundation laid by prior reviews addressing individual aspects of alpaca reproductive biology (4, 19, 20), this review offers an integrated account of both female and male reproductive physiology spanning all major ART modalities within a unified mechanistic framework. Bringing these two physiological systems together in a single synthesis is essential at this stage of the field’s development, since female and male reproductive events in alpacas are mechanistically linked rather than independent, and a combined view is what allows the shared regulatory pathways connecting them to come into focus. This review synthesizes current knowledge of alpaca reproductive anatomy, follicular and neuroendocrine physiology, seminal plasma biochemistry, semen cryopreservation, and ART development to identify critical knowledge gaps and prioritize future research. Particular emphasis is placed on the mechanistic determinants of ART efficiency and translating laboratory findings into field-applicable protocols for genetic improvement in alpacas globally.

## 2 Female reproductive anatomy

The female reproductive tract of the alpaca is highly specialized and exhibits structural characteristics with important implications for reproductive management and ARTs. Externally, the perineal region features a vertically oriented vulva with closely apposed labia that maintain a tightly sealed configuration, minimizing ascending contamination (21, 22). Female alpacas exhibit no consistent external indicators of reproductive status, such as vulvar hyperemia or mucosal discharge, necessitating ultrasonographic monitoring for accurate determination of ovarian and uterine condition (21).

Internally, the vagina is relatively long and narrow, extending cranially to the cervix, which measures approximately 2 to 5 cm in length and 2 to 4 cm in diameter and contains either two to three cartilaginous rings or a single spiral fold with multiple ring-like turns, as documented by vaginoscopy and transrectal ultrasonography (22–24). Despite this complex intraluminal architecture, the anatomical proximity of the cervix to the rectum permits reliable transcervical access under both estrogen- and progesterone-dominant hormonal conditions, facilitating AI, uterine flushing, and embryo transfer (21). The fibroelastic penis of the alpaca, equipped with a cartilaginous tip, achieves full intracornual intromission during natural mating, producing widespread epithelial abrasion throughout the cervix and uterine horns; the physiological significance of this copulatory wounding is addressed in section 2.4 (25, 26).

The uterus is bicornuate, with a short body of approximately 3 to 5.5 cm and two asymmetrically developed horns exhibiting consistent and pronounced left-horn dominance (21, 22). This asymmetry is present in nulliparous females and female fetuses, indicating a developmental rather than gestational origin, and is associated with differential vascularization (7). More than 98% of pregnancies are established and carried to term in the left uterine horn regardless of ovulation laterality, requiring embryos of right-ovary origin to undergo transcornual migration prior to implantation (27, 28). The oviducts are long and tortuous, connected to the uterine horns via a well-defined sphincteric papilla that prevents retrograde flushing; the left oviduct exhibits histoarchitectural differences from the right in epithelial cell proportions and luminal diameter, reflecting its predominant functional role in gamete and embryo transport (29).

### 2.1 Ovarian follicular dynamics and neuroendocrine control of ovulation

The first systematic characterization of alpaca ovarian function was reported by San-Martin et al. (30), who established that ovulation occurs as early as 26 h after mating. Subsequent adoption of transrectal ultrasonography and sensitive immunoassay platforms enabled researchers across South America, North America, Australia, and Europe to characterize follicular kinetics with precision not previously achievable (10, 11, 31, 32).

#### 2.1.1 Follicular wave dynamics: continuous activity without cyclic ovulation

The ovarian follicular dynamics of alpacas differ fundamentally from those of spontaneously ovulating livestock. In cattle, sheep, and goats, follicular waves emerge within a progesterone-dominated luteal phase that ultimately yields a spontaneous preovulatory LH surge. In alpacas, by contrast, follicular wave activity proceeds continuously in the absence of progesterone in non-mated females, since these are obligate induced ovulators requiring a copulatory or pharmacological stimulus to trigger LH release and follicular rupture (10, 30). This strategy results in prolonged periods of follicular estradiol secretion and behavioral receptivity that are not interrupted by spontaneous ovulation, rendering the ovarian environment inherently dynamic and temporally less predictable than in cyclic species.

Each follicular wave proceeds through three phases, recruitment of a cohort of small antral follicles (3 to 5 mm in diameter); selection, during which one follicle acquires dominance; and a static or plateau phase, during which the dominant follicle reaches 7 to 12 mm and suppresses subordinate follicular growth on both ovaries through estradiol-mediated FSH suppression (22, 32, 33). Mean dominant follicle growth rates have been estimated at approximately 0.43 mm/day (32). In the absence of mating or pharmacological induction, the dominant follicle undergoes atresia and is replaced by the subsequent wave, with inter-wave intervals of 10 to 22 days exhibiting considerable intra- and interindividual variability (10, 31). A distinctive feature of alpaca follicular dynamics is wave overlap, whereby a new follicular cohort emerges before regression of the preceding dominant follicle is complete, creating a transitional period of bidirectional follicular activity that complicates ultrasonographic interpretation in ART contexts (32).

The follicle size threshold for ovulatory responsiveness has direct implications for ARTs. Females with dominant follicles of 7 mm or greater consistently ovulate in response to mating, exogenous GnRH, or intramuscular ovulation-inducing factor (OIF) administration (31, 34–36), whereas females with follicles below this threshold or with regressing dominant follicles exhibit substantially reduced ovulatory and pregnancy rates (31, 35). This threshold motivates the development of follicular synchronization protocols designed to ensure an appropriately sized dominant follicle at a defined time prior to ART procedures.

#### 2.1.2 Neuroendocrine events of ovulation: the LH surge and copulatory stimuli

Copulation initiates a rapid neuroendocrine cascade culminating in the preovulatory LH surge and follicular rupture. The LH surge is detectable in peripheral blood within 15 to 40 min of mating onset, reaches peak concentrations between 2 and 3 h post-copulation, and returns to basal levels within 4 to 12 h (31, 37). Ovulation averages approximately 30 h post-mating (range: 24 to 48 h), is largely independent of follicular size within the responsive range, and occurs with comparable frequency on both ovaries without preferential laterality (31, 36). Multiple ovulations have been documented in 5 to 15% of matings, predominantly reflecting aspiration of a second large follicle from the contralateral ovary (38).

The preovulatory LH surge initiates coordinated intrafollicular events including oocyte nuclear maturation from dictyate arrest through metaphase II completion, cumulus cell expansion mediated by hyaluronic acid deposition, increased follicular wall vascularization detectable by color Doppler ultrasonography, and ultimately follicular rupture with cumulus-oocyte complex (COC) release into the ovarian infundibulum (31, 39). Estradiol concentrations remain stable for approximately 18 h post-mating before declining significantly by 48 h, reflecting the transition from follicular to luteal steroidogenesis (40).

#### 2.1.3 Beta-nerve growth factor as the ovulation-inducing factor in seminal plasma

The molecular identity of OIF in camelid seminal plasma represents one of the most significant advances in comparative reproductive endocrinology of recent decades. Its existence was first demonstrated by Paolicchi et al. (41), who showed that intrauterine administration of alpaca seminal plasma stimulated pituitary LH secretion. Adams et al. (42) subsequently established that intramuscular injection of seminal plasma reliably induced preovulatory LH surge and ovulation, confirming a systemic endocrine rather than local paracrine mechanism. Ratto et al. (39) achieved definitive molecular characterization of OIF as the beta subunit of nerve growth factor (*β*-NGF) through size-exclusion chromatography, mass spectrometry, X-ray crystallography, and bioactivity assays; the purified fraction exhibited a molecular mass of 13.221 kDa, shared 12 to 23 amino acid sequence identity with human, porcine, bovine, and murine *β*-NGF, and induced TrkA upregulation in neuronal models with potency equivalent to recombinant NGF. Independent proteomic confirmation was provided simultaneously by Kershaw-Young et al. (43), who identified *β*-NGF as the single most abundant protein in alpaca seminal plasma.

Following intrauterine semen deposition, *β*-NGF is absorbed through the endometrial mucosa into systemic circulation; transmucosal absorption is accelerated by the epithelial disruption caused by the cartilaginous penile tip during intracornual copulation (25, 26, 44). Circulating *β*-NGF acts at the anterior pituitary, which lacks blood–brain barrier protection and is directly accessible to circulating neurotrophin, and at the hypothalamus, where it activates kisspeptin neurons in the preoptic area and arcuate nucleus via TrkA receptor binding, driving pulsatile GnRH release and consequently the preovulatory LH surge (39, 45–47). Pre-treatment with the GnRH antagonist cetrorelix completely abolishes the LH surge and ovulation induced by both natural mating and exogenous seminal plasma, confirming GnRH receptor activation as an obligate step in the *β*-NGF-mediated ovulatory pathway (48).

Beyond ovulation induction, *β*-NGF exerts luteotrophic effects that distinguish it from conventional pharmacological agents. Females receiving seminal plasma-derived *β*-NGF at induction exhibit significantly larger corpus luteum (CL) diameter, greater vascular perfusion assessed by color Doppler ultrasonography, and higher peripheral progesterone concentrations between days 4 and 8 post-induction relative to GnRH or saline controls (49, 50). These effects are mechanistically underpinned by increased mRNA expression of steroidogenic acute regulatory protein (StAR), CYP11A1, and 3*β*-HSD within luteal tissue, confirming that *β*-NGF upregulates the transcriptional machinery of luteal steroidogenesis in addition to promoting luteal vascularization (51). TrkA receptor immunoreactivity has been localized to luteal cells and to granulosa and theca cells of dominant follicles in llamas and cattle, providing the cellular substrate for these direct ovarian actions (46, 52). Phylogenetically, OIF/*β*-NGF with ovulatory bioactivity has been identified in rabbits, koalas, domestic cats, vicunas, and Bactrian camels, and detected at lower concentrations in cattle, horses, and pigs, suggesting that neurotrophin-mediated ovulation induction may be phylogenetically widespread rather than a camelid-specific adaptation (44, 53).

Comparative evidence from other camelid species underscores the centrality of seminal *β*-NGF in induced ovulation across the family Camelidae. The ovulation-inducing capacity of seminal plasma was, in fact, first documented not in South American camelids but in the Bactrian camel, where intravaginal administration of seminal plasma alone induced ovulation in 87% of treated females, establishing the paradigm later extended to llamas and alpacas (54). Direct cross-species comparison of OIF bioactivity in the seminal plasma of llamas, alpacas, and bulls confirmed that camelid seminal plasma induces a substantially greater and more consistent ovulatory response than bovine seminal plasma, despite low-level OIF/*β*-NGF bioactivity being detectable in both induced and spontaneous ovulators (55). These findings position the Old World camel not as a divergent outgroup but as the species in which the induced-ovulation mechanism governing alpaca reproduction was first mechanistically defined, underscoring that camelid-specific, rather than bovine- or ovine-derived, physiological benchmarks should guide the interpretation of alpaca ovulation-induction protocols.

From an ARTs perspective, current GnRH analogue and hCG protocols induce LH surges of shorter duration and lower peak magnitude than natural mating and do not replicate the luteotrophic support of seminal *β*-NGF (31). The development of recombinant or highly purified llama-derived *β*-NGF preparations suitable for intramuscular administration therefore represents a high-priority translational objective, particularly for synchronization and superstimulation protocols where optimal CL function is critical for embryo viability.

**Figure 1:**
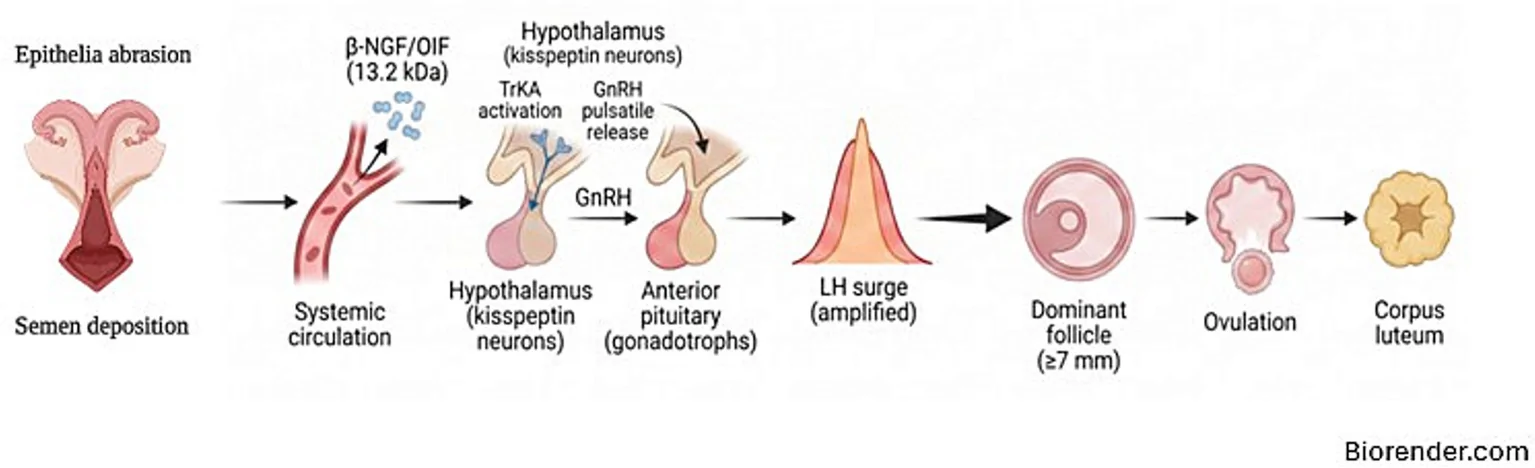
Schematic representation of the *β*-nerve growth factor (*β*-NGF)/ovulation-inducing factor (OIF)-mediated neuroendocrine cascade driving the preovulatory LH surge and luteal steroidogenesis in the alpaca (*Vicugna pacos*). Following intracornual semen deposition, *β*-NGF/OIF (13.2 kDa) is absorbed transmucosally into systemic circulation, a process facilitated by epithelial abrasion from the cartilaginous penile tip. Circulating *β*-NGF activates TrkA receptors on kisspeptin neurons of the hypothalamus (preoptic area and arcuate nucleus), driving pulsatile GnRH secretion into the hypothalamo-hypophyseal portal system. At the anterior pituitary, GnRH receptor activation on gonadotroph cells elicits an amplified preovulatory LH surge that induces ovulation of the dominant follicle (≥7 mm) and subsequent corpus luteum formation. Direct TrkA-mediated actions of *β*-NGF on luteal, granulosa, and theca cells further upregulate the steroidogenic machinery, resulting in enhanced corpus luteum vascularization and progesterone output relative to conventional GnRH analogue protocols. *β*-NGF, beta-nerve growth factor; OIF, ovulation-inducing factor; GnRH, gonadotropin-releasing hormone; LH, luteinizing hormone; TrkA, tropomyosin receptor kinase A. Created in BioRender. Landinez-Aponte, J. (2026) https://BioRender.com/ovjizdf.

The neuroendocrine cascade through which seminal *β*-NGF drives the preovulatory LH surge is summarized in Figure 1. Following transmucosal absorption of *β*-NGF/OIF (13.2 kDa) from the endometrial mucosa into systemic circulation, a process accelerated by epithelial abrasion from the cartilaginous penile tip, circulating neurotrophin sequentially activates kisspeptin neurons in the hypothalamus via TrkA receptor binding, drives pulsatile GnRH release to the anterior pituitary, and stimulates amplified LH secretion by gonadotroph cells, ultimately inducing ovulation of the dominant follicle and corpus luteum formation. The direct luteotrophic actions of *β*-NGF on TrkA-positive luteal, granulosa, and theca cells, which underpin the enhanced steroidogenic capacity of the resulting corpus luteum, are also represented.

### 2.2 Corpus luteum formation, progesterone dynamics, and Luteolysis

Following ovulation, the CL becomes detectable by transrectal ultrasonography from approximately day 4 post-ovulation as a medium-echogenicity structure, often with a central fluid-filled cavity of 3 to 8 mm (11). Peripheral progesterone concentrations begin to rise measurably on days 4 to 6 post-mating, reaching peak values between days 7 and 9; concentrations exceeding 2 ng/mL (6.4 nmol/L) are considered indicative of a fully functional CL (40, 56). The CL reaches its maximum structural size of 10 to 15 mm concurrently with peak progesterone secretion, with luteolysis functionally complete by approximately days 10 to 12 post-mating and behavioral receptivity returning 12 to 14 days after mating in the absence of conception (56, 57).

Luteolysis is mediated by endometrial prostaglandin F2α (PGF2α) secreted in a pulsatile pattern during days 7 to 11 post-mating, coincident with maximal endometrial COX-2 expression (56, 58). A functionally important asymmetry governs luteolytic influence, the right uterine horn exerts luteolytic activity exclusively on the ipsilateral CL via a local utero-ovarian countercurrent mechanism, whereas the left horn can induce luteolysis of corpora lutea on either ovary through both local and systemic pathways, attributed to differential vascular architecture and venous drainage patterns (59, 60). Unlike cattle and sheep, oxytocin does not appear to participate in the luteolytic process in camelids (58). Short luteal phases, associated with luteinization of non-ovulated dominant or atretic follicles and characterized by approximately 5-day functional lifespan, may contribute to elevated early embryonic loss rates in this species (34, 61).

**Figure 2:**
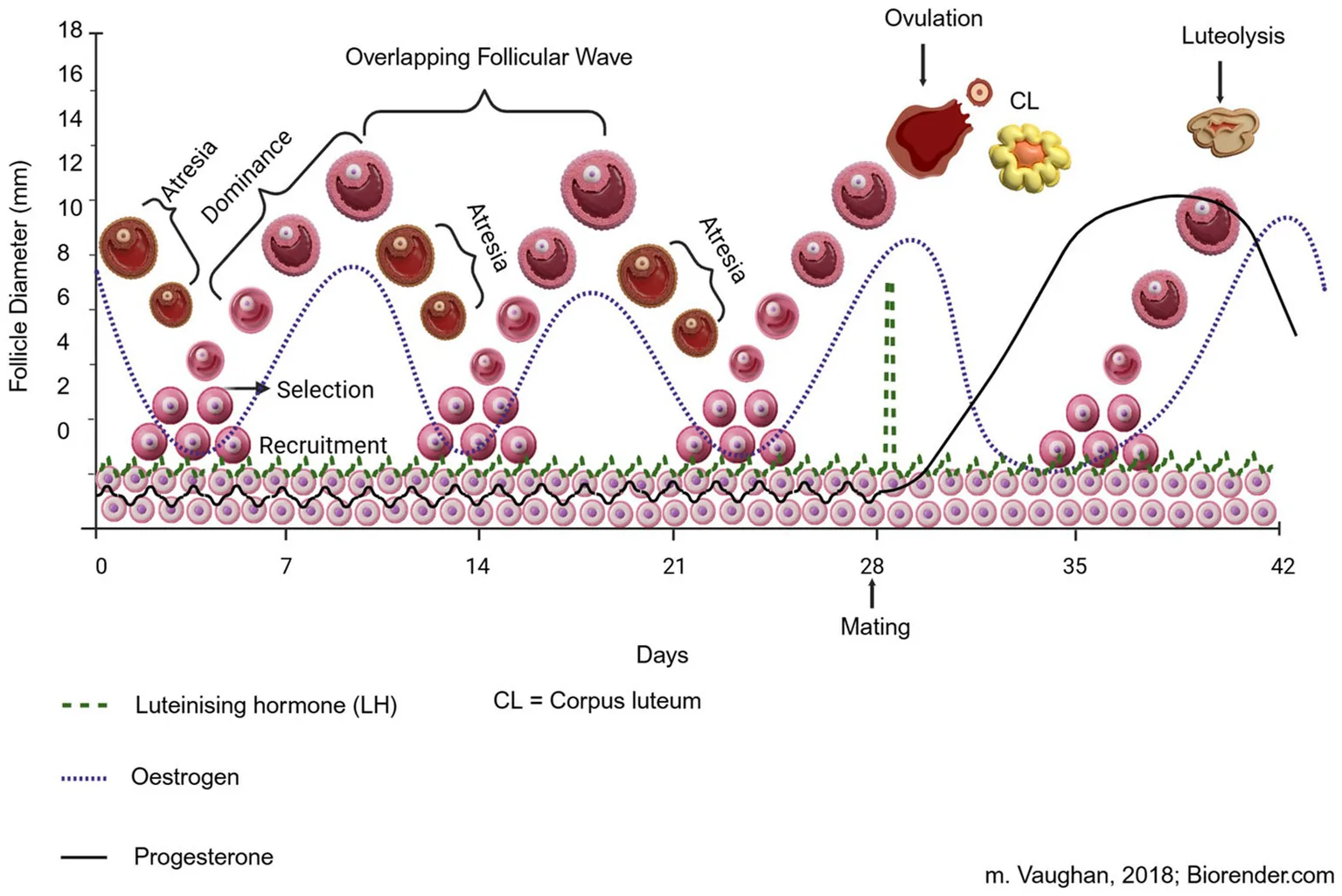
Schematic representation of continuous follicular wave dynamics, hormonal profiles, and luteal events across a representative 42-day reproductive cycle in the alpaca (*Vicugna pacos*). Follicular waves recur at approximately 7-day intervals in a continuous, non-seasonal pattern, with each wave progressing through sequential phases of recruitment, selection, dominance, and atresia. Mating on day 28 triggers a pulsatile LH surge that induces ovulation of the dominant follicle, followed by corpus luteum (CL) formation, a progressive rise in peripheral progesterone concentrations peaking between days 7 and 9 post-mating, and functional luteolysis by approximately days 10 to 12 in the absence of conception. Oestrogen concentrations fluctuate in parallel with dominant follicle growth across successive waves. A new follicular wave resumes following luteolysis, illustrating the uninterrupted follicular activity that characterizes this induced-ovulator species. LH, luteinizing hormone; CL, corpus luteum. Created in BioRender. Landinez-Aponte, J. (2026) https://BioRender.com/q4a5ogp.

The temporal integration of follicular wave dynamics, ovulation, corpus luteum formation, progesterone secretion, and luteolysis described in sections 2.1 and 2.2 is illustrated schematically in Figure 2. The diagram depicts the continuous, non-seasonal pattern of overlapping follicular waves in the alpaca over a representative 42-day period, with sequential phases of recruitment, selection, dominance, and atresia recurring at approximately 7-day intervals. Mating-induced ovulation triggers the LH surge, followed by CL establishment, a progesterone rise peaking between days 7 and 9 post-mating, and functional luteolysis by approximately days 10 to 12 in the absence of conception, when a new follicular wave resumes (62). This integrated endocrine and follicular profile underscores the obligate induced-ovulation mechanism and the continuous follicular availability that collectively define the reproductive strategy of this species and govern the timing requirements of all ART interventions.

### 2.3 Ovarian synchronization strategies for assisted reproductive technologies

Effective implementation of ARTs in alpacas requires precise control of follicular wave emergence to ensure a recruitable follicular cohort at a defined time point relative to ovarian stimulation. Because the dominant follicle strongly suppresses subordinate follicular growth, its elimination is a prerequisite for reliable follicular wave synchronization. This can be achieved pharmacologically through GnRH-induced ovulation or mechanically through ultrasound-guided follicular ablation; both interventions, designated day 0, are followed by consistent emergence of a new follicular wave approximately 48 h later in alpacas and llamas, providing a predictable window for initiating superstimulatory FSH treatment (7, 31, 35).

Superstimulation protocols evaluated in alpacas have employed porcine FSH (pFSH) at doses of 100 to 200 mg in decreasing-dose regimens over 2 to 4 days, or equine chorionic gonadotropin (eCG) at 700 to 1,000 IU as a single injection, both initiated 48 to 72 h after follicular wave emergence (31, 63). Under optimized conditions, an average of 10 to 15 follicles per ovary can be recruited (63); however, reported ranges extend from 0 to more than 25 follicles per female within the same protocol, reflecting the combined influence of genetic background, nutritional status, baseline antral follicle count, and the hormonal environment at wave induction (31, 63). Data from our laboratory indicate that initiation of superstimulation with 200 mg of NIH-pFSH-P1 at 48 h after GnRH-induced ovulation of the dominant follicle (≥7 mm in diameter) consistently supports development of at least 12 follicles per ovary on average, of which 74% were amenable to aspiration. Using the LOPU approach, a mean of 6 oocytes per donor was recovered at a 60% oocyte recovery rate, with more than 50% of collected oocytes classified as excellent morphological quality (Grades I and II), demonstrating that the combination of GnRH synchronization and FSH superstimulation not only drives robust follicular recruitment but supports the recovery of a developmentally competent oocyte cohort suitable for downstream assisted reproductive procedures (64).

## 3 Male reproductive anatomy and Spermatology

The male reproductive tract of the alpaca is characterized by anatomical specializations that depart markedly from conventional domestic ruminants, with direct consequences for semen biology and the development of ARTs. The scrotum is non-pendulous, firmly positioned at the ischial arch approximately 5 to 9 cm ventral to the anus, in fundamental contrast to the pendulous configuration of bulls and rams in which passive convective cooling mediates thermoregulation (21, 65, 66). The testes are small and elliptical, averaging approximately 17 g per gonad, with pronounced interindividual variation attributable to nutritional plane, genetic background, altitude, and season; neither testosterone concentration nor ultrasonographic biometrics alone reliably predicts individual fertility, underscoring the multifactorial nature of male reproductive potential (67). The epididymis is compact and firmly adherent to the testicular parenchyma, composed of caput, corpus, and cauda regions, with sympathetic and peptidergic neural pathways modulating luminal fluid composition, smooth muscle contractility, and sperm transport kinetics (65, 66, 68).

The phylogenetically consistent absence of seminal vesicles across all Camelidae constitutes the most consequential anatomical feature of the male tract, fundamentally altering seminal plasma biochemistry and constraining conventional cryopreservation approaches, as detailed in section 3.3 (23, 65). The prostate gland, the sole well-differentiated accessory sex gland, is a small H-shaped structure opposed to the dorsolateral pelvic urethra, with multiple ducts opening into the colliculus seminalis (65, 66). The paired bulbourethral glands represent the principal source of mucin 5B (MUC5B), the gel-forming mucin responsible for the characteristic high viscosity of alpaca seminal plasma (12, 69). The penis is fibroelastic, maintained intrapreputially via a pre-scrotal sigmoid flexure, with a total length of 35 to 45 cm and a distal cartilaginous tip that constitutes the primary agent of widespread epithelial abrasion throughout the female reproductive tract during natural mating (21, 25, 26). This copulatory wounding facilitates transmucosal absorption of seminal *β*-NGF and potentiates the neuroendocrine ovulatory cascade described in section 2.1.3 (9, 25, 26, 69, 70). Complete fusion of the penile epithelium with the preputial mucosa at birth is universal; androgenic stimulation drives progressive preputial detachment over several years, with functional breeding capacity typically attained at approximately 3 years of age despite earlier onset of spermatogenic activity (21, 65).

### 3.1 Testicular ontogeny and spermatogenesis

Spermatogenesis in the alpaca follows a developmental timeline considerably more protracted than in conventional livestock. At birth, the testes contain undifferentiated Leydig cells and gonocytes within seminiferous cords lacking a defined lumen. A protracted infantile phase extends from approximately 2 to 11 months of age during which germ cell differentiation remains quiescent (21, 66, 71). Tubulogenesis commences at approximately 12 months, and the first morphologically identifiable spermatozoa are detectable within the caput epididymidis at approximately 18 months of age in approximately 50% of males, with universal prevalence by 22 months (21, 66). Histologically complete spermatogenesis is established between 18 and 24 months, with testicular volume reaching a plateau at approximately 30 months coinciding with maximal reproductive output (71). Despite spermatogenic competence being achievable by 2 years, productive use of males is conventionally deferred until 3 years (65, 67).

Testosterone secretion from interstitial Leydig cells governs spermatogenesis, secondary sexual differentiation, and preputial maturation. Pharmacological challenge with exogenous hCG demonstrates a marked increase in testicular steroidogenic responsiveness between 13 and 14 months of age, consistent with Leydig cell population expansion (71). Mean plasma testosterone concentrations of 2.47 ± 0.31, 8.45 ± 1.53, and 22.66 ng/mL have been reported in young, intermediate, and adult Huacaya alpaca males, respectively, confirming that maximum Leydig cell output coincides with full tubular spermatogenesis at 24 months (65–67). Seasonal fluctuations in testicular volume documented in alpacas maintained under Northern European photoperiodic conditions suggest that ambient light cycle and temperature may modulate testicular hemodynamics even in populations not classically considered seasonally photoperiodic, highlighting the need for systematic evaluation of environmental effects on male fertility in temperate production systems (67).

The cellular organization of the seminiferous epithelium conforms to the conserved mammalian spermatogenic sequence, with all germ cell classes supported within a Sertoli cell scaffold that mediates the blood-testis barrier, coordinates meiosis and spermiogenesis, and secretes androgen-binding protein and inhibin B (65, 72). Testicular fine needle aspiration (TFNA) has been validated as a minimally invasive modality for cytological characterization of seminiferous epithelial populations without measurable adverse effects on sperm quality or testicular architecture, establishing its utility for male infertility investigation and pre-breeding soundness evaluation (73).

### 3.2 Beta-nerve growth factor in testicular biology

*β*-NGF functions not only as the seminal OIF described in section 2.1.3 but also as an endogenous regulator of testicular development. Immunohistochemical and quantitative PCR analyses of NGF expression across postnatal testicular developmental periods reveal significant age-dependent differences between neonatal animals and those at 12 and 24 months, with concentrated expression precisely during the prepubertal to early pubertal transition when gonocytes must exit mitotic quiescence and initiate spermatogenic differentiation (74). Co-localization of *β*-NGF with its high-affinity receptor TrkA in the midpiece of ejaculated llama spermatozoa raises the possibility of autocrine modulation of mitochondrial activity and flagellar motility, a pathway requiring direct functional validation in alpaca spermatozoa (75); given the close evolutionary relationship between alpacas and llamas, the colocalization of *β*-NGF and TrkA in the sperm midpiece is likely conserved in alpacas spermatozoa. Resolving the intratesticular and post-testicular functions of *β*-NGF represents a research priority given its potential as both a diagnostic biomarker of testicular maturation and a target for interventions aimed at improving semen quality in subfertile males.

### 3.3 Seminal plasma: biochemistry, viscosity determinants, and sperm function

The distinctive biochemical profile of alpaca seminal plasma is inextricably linked to the absence of vesicular glands across the family Camelidae. In bulls and rams, vesicular glands account for more than 50% of total ejaculate volume and provide fructose, citric acid, and semenogelin proteins that mediate reversible seminal coagulation and subsequent liquefaction (76). In alpacas, secretory activity derives exclusively from the comparatively small prostate and bulbourethral glands, yielding a characteristically low-volume ejaculate of 1 to 2 mL in which seminal plasma constitutes approximately 85% of total volume and spermatozoa only 15%, a proportion markedly inverted relative to rams and bulls (23, 69, 77, 78). Biochemical profiling confirms substantially lower fructose and citric acid concentrations than in ram and bull semen, while total protein concentrations are broadly comparable (69, 78, 79). Cross-species proteomic analysis across seven mammalian species identified nucleobindin-1 and RSVP14 as universally conserved seminal plasma proteins, situating the alpaca proteome within the conserved mammalian framework while highlighting phylogenetically distinctive features of its glandular complement (80).

For several decades, glycosaminoglycans (GAGs) were the primary molecular candidates proposed to account for alpaca seminal plasma viscosity, given the established role of the bulbourethral gland as a GAG synthesis site and the known capacity of GAG polymers to impart viscoelastic properties to biological fluids (81, 82). Biochemical characterization confirmed all five major GAG classes in alpaca seminal plasma, with total concentrations approximately 15-fold higher than in ram seminal plasma and a statistically significant positive correlation between keratan sulphate concentration and thread-test viscosity (43). However, direct experimental evaluation through targeted enzymatic degradation with keratanase, hyaluronidase, and chondroitinase ABC produced viscosity reductions statistically indistinguishable from spontaneous reduction in untreated controls over identical incubation periods, refuting a primary causal role for GAGs in alpaca ejaculate rheology (12). While GAGs do not constitute the structural backbone of seminal plasma viscoelasticity, they are not without functional relevance. GAGs modulate sperm capacitation dynamics, the acrosome reaction, and sperm-oviductal adhesion in multiple mammalian species, and the seminal lectin SL15 mediates binding of ejaculated spermatozoa to N-acetylgalactosamine residues on the oviductal epithelial surface, a carbohydrate-recognition interaction absent from epididymal spermatozoa (83–86).

The protein-based viscosity hypothesis is supported by broad and consistent efficacy of multiple mechanistically distinct proteases. Papain (*Carica papaya* cysteine protease; 0.1 mg/mL, 37 °C, 20 min) completely eliminated alpaca seminal plasma viscosity while preserving sperm motility, viability, acrosomal integrity, and DNA integrity (12). Proteinase K achieved equivalent liquefaction at 1 mg/mL within 60 min (12), and earlier work had established comparable viscosity-reducing activity for collagenase, trypsin, and fibrinolysin, though not uniformly sperm-safe (13). Crucially, the cysteine-specific mechanism of papain, which hydrolyzes proteoglycan core proteins while leaving GAG side chains intact, provided direct mechanistic evidence that protein scaffolding rather than GAG polymers constitutes the structural backbone of seminal plasma viscoelasticity.

Unpublished data from our laboratory further demonstrate that the sequential papain-E-64 protocol is compatible with the complete *in vitro* fertilization pipeline. Electroejaculated semen subjected to this treatment achieved complete viscosity elimination, enabling standard sperm processing otherwise precluded by the gel-like consistency of untreated ejaculates. *In vitro*-matured oocytes recovered from live donors via laparoscopic ovum pick-up (LOPU) served as the fertilization substrate, integrating two of the most technically demanding ARTs in this species within a single experimental framework. Fertilization outcomes were unaffected by protease treatment, confirming that liquefaction preserves sperm fertilizing capacity at the level of oocyte penetration. Treated spermatozoa additionally exhibited enhanced kinematic performance relative to untreated controls, including higher linearity (LIN, %), straight-line velocity (VSL, μm/s), straightness (STR, %), and wobble (WOB, %), suggesting that viscosity elimination relieves a biophysical constraint on flagellar motion not fully reflected by conventional motility endpoints. These results establish the papain-E-64 protocol as the first viscosity-reduction strategy validated across the complete male-to-embryo reproductive chain in alpacas (unpublished data).

Definitive identification of the principal viscosity-determining protein was achieved through comparative iTRAQ mass spectrometry of seminal plasma pools stratified by viscosity phenotype. Mucin 5B (MUC5B) was identified at greater than fivefold higher abundance in high-viscosity relative to low-viscosity samples (69). MUC5B belongs to the gel-forming mucin family and is structurally defined by a large O-glycosylated central tandem repeat domain flanked by cysteine-rich von Willebrand factor D-like domains enabling disulfide bond-mediated polymerization into high-molecular-weight viscoelastic gel networks; it is this supramolecular polymerization capacity that confers the characteristic rheological behavior of alpaca seminal plasma (87). Its proteomic identification is anatomically coherent with the established role of the bulbourethral gland as the primary secretory source of seminal plasma viscosity (69, 88, 89). Dedicated shotgun proteomic pipelines incorporating density-gradient mucin enrichment, reductive carboxymethylation, and sequential trypsin digestion have been proposed for definitive glycoform characterization (90).

Despite MUC5B-driven viscosity constituting the primary ART barrier, seminal plasma also encodes an indispensable sperm-protective function. A minimum residual concentration of approximately 10% seminal plasma is required to maintain motility, acrosomal integrity, and viability during processing and post-thaw incubation; complete removal has consistently deleterious effects on sperm function (83, 91). Supplementation of cryopreserved vas deferens-derived spermatozoa with 10% autologous seminal plasma at post-thaw significantly improved motility, morphological normality, and membrane integrity, providing direct evidence for a sperm-protective role of seminal plasma proteins independent of their viscosity-associated limitations (83). This paradoxical dual role, as both the primary impediment to ART processing and an indispensable biological medium for sperm function, defines the central translational challenge of alpaca semen handling.

The high viscosity of camelid semen is not unique to alpacas. Dromedary camel ejaculate is similarly gelatinous and originates from the same glandular source, since Old World camelids also lack seminal vesicles and rely on bulbourethral and prostatic secretions for seminal plasma production (92). The protein driver of this viscosity, however, appears to differ between the two lineages. In dromedary camels, liquefaction over 18 to 24 h coincides with proteolytic cleavage of a 26 kDa protein into 24.55 and 22.07 kDa fragments identified as *β*-NGF, implicating the same neurotrophin responsible for ovulation induction in the formation of the semen coagulum, a dual functional role not yet described for alpaca *β*-NGF (93). This divergence, MUC5B as the principal viscosity determinant in alpacas versus a *β*-NGF-associated coagulum in camels, suggests that despite a shared glandular origin and a shared reliance on *β*-NGF/OIF for ovulation induction, the biochemical basis of ejaculate viscoelasticity may have diverged within Camelidae, a distinction with direct implications for selecting species-appropriate liquefaction strategies in ART protocols. Comprehensive mass spectrometric profiling of alpaca seminal plasma has catalogued 89 proteins, of which 28 share significant sequence homology with seminal plasma proteins functionally associated with sperm membrane stabilization, capacitation suppression, zona pellucida binding, and the acrosome reaction in other species (69). These include heparin-binding spermadhesins (clusterin, lactoferrin, ELSPBP1), cysteine-rich secretory protein 1 (CRISP1), fibronectin type II domain-containing proteins, and glycan-processing enzymes. Among these, phosphatidylethanolamine-binding protein 1 (PEBP1) is particularly noteworthy; it was identified at approximately twofold higher abundance in low-viscosity seminal plasma, correlates with superior fertilizing ability in bulls through Raf kinase modulation of premature capacitation, and has been identified in alpaca oviductal fluid as a decapacitation-associated factor contributing to sperm reservoir maintenance (94, 95). PEBP1 represents a molecularly tractable candidate for investigation as both a predictive biomarker for cryopreservation outcomes and a target for seminal plasma reconstitution strategies.

### 3.4 Cryopreservation of alpaca spermatozoa

The cryopreservation of alpaca spermatozoa has remained one of the most technically challenging unsolved problems in South American Camelid reproductive biotechnology, with MUC5B-driven seminal plasma viscosity as the foundational obstacle. By physically preventing uniform penetration of cryoprotective extenders into the sperm-plasma matrix, viscosity impairs cryoprotectant-membrane contact and consistently yields post-thaw progressive motility averaging 15 to 25% across camelid studies (13, 96). The initial systematic investigation by Santiani et al. (96) established that skim milk and egg yolk extenders containing ethylene glycol yielded the highest post-thaw acrosomal integrity, providing the first evidence-based cryoprotectant selection framework.

To circumvent the viscosity constraint, subsequent investigations redirected efforts toward epididymal and vas deferens-derived spermatozoa, which lack the viscous seminal plasma fraction and are directly amenable to extender dilution. Morton et al. (97) established that a lactose-based diluent with pellet freezing yielded significantly higher post-thaw motility than citrate- or Tris-based alternatives for epididymal spermatozoa; Morton et al. (98) subsequently found that 3% glycerol with 1% Equex STM supplementation improved post-thaw acrosomal integrity, though motility remained below 25%. Mamani-Mango et al. (99) provided the first direct evidence that cryopreserved epididymal alpaca spermatozoa retain fertilization capacity *in vitro*, albeit at low and variable rates.

The critical advance enabling cryopreservation of ejaculated semen was achieved by Kershaw et al. (100), who established the sequential papain and E-64 inhibitor protocol as the first clinically applicable liquefaction procedure compatible with downstream cryopreservation. Papain treatment at 0.1 mg/mL for 20 min at 37 °C completely eliminates seminal plasma viscosity, and subsequent inhibition with E-64 (10 μM, 5 min, 37 °C) halts proteolytic activity, preventing progressive sperm membrane damage during freezing processing; papain-plus-E-64-treated spermatozoa exhibited significantly higher total motility at post-chill, immediately post-thaw, and 1 h post-thaw relative to untreated controls (100). Stuart et al. (101) subsequently conducted comprehensive optimization of the full cryopreservation pipeline for papain-treated ejaculated spermatozoa, achieving post-thaw total motility values of up to 45% using a lactose-based diluent with 5% egg yolk in 0.25 mL straws, and confirmed pregnancy following deep uterine AI with cryopreserved papain-treated spermatozoa. These milestones constitute the current clinical benchmark for ejaculated alpaca semen cryopreservation.

Aisen et al. (83) demonstrated the restorative utility of seminal plasma reconstitution, confirming that 10% autologous seminal plasma supplementation at post-thaw significantly improved motility, morphological normality, and membrane integrity of cryopreserved vas deferens-derived spermatozoa. Antioxidant strategies represent a complementary avenue. Levano et al. (102) reported that atomized black maca (*Lepidium meyenii*) added to the freezing medium of epididymal spermatozoa of alpacas at 20 mg/mL improved post-thaw motility, viability, plasma membrane integrity, mitochondrial function, and reactive oxygen species production in epididymal spermatozoa. The most recent *in vivo* comparison by Huamani et al. (103) reported pregnancy rates of 33.3 and 22.2% with Biladyl B and AndroMed extenders, respectively, confirming feasibility but underscoring the gap relative to conventional livestock performance. The endogenous protease system responsible for spontaneous *in vivo* seminal plasma liquefaction during the postcopulatory interval remains unidentified and represents an important unresolved question in camelid spermatology (69).

Spermatogonial stem cells (SSCs) represent an underutilized avenue for germline preservation. Valdivia et al. (72) demonstrated for the first time that testicular tissue fragments and isolated SSCs from adult alpaca testes 24 h post-mortem can be cryopreserved using media supplemented with atomized black maca, with SSC identity confirmed by flow cytometry and post-thaw viability maintained in more than 50% of specimens. Integration of SSC banking and surrogate sire transplantation into national alpaca genetic improvement programs represents a substantive opportunity for preserving high-fiber genotypes disproportionately vulnerable to attrition through conventional selection practices (67, 72).

## 4 Assisted reproductive technologies

The implementation of ARTs in alpacas is constrained by the species-specific physiological barriers detailed in the preceding sections. Collectively, obligate induced ovulation, continuous follicular wave dynamics without spontaneous cyclic regulation, MUC5B-driven seminal plasma viscosity, and low oocyte developmental competence under *in vitro* conditions define a technical landscape fundamentally distinct from that of conventional livestock. Current ART modalities under investigation, including AI, MOET, LOPU-based IVEP, and ICSI, have achieved proof-of-concept outcomes but none has reached the threshold of standardized, field-validated commercial application. The following sections critically evaluate the state of each modality against this backdrop.

### 4.1 Artificial insemination

The first documented AI success in alpacas was reported by Fernandez-Baca and Novoa (104), who achieved cria births following insemination with fresh semen collected by electroejaculation from paco-vicuna hybrid males, establishing technical feasibility. In the same year, Calderon et al. (105) demonstrated the critical role of ovulation induction strategy and insemination timing, reporting pregnancy rates of 100 and 83.3% at 35 to 45 and 46 to 52 h post-vasectomized-male induction, respectively, compared with substantially lower rates in hCG-treated females. Bravo et al. (77) subsequently conducted the first systematic AI study using semen collected by artificial vagina, achieving an overall pregnancy rate of 68% with fresh undiluted semen deposited by laparoscopy or transcervical route following hCG-induced ovulation. Bravo et al. (106) established the first evidence-based sperm dose threshold, deep intrauterine insemination with 8 million spermatozoa per mL yielded 66.7% pregnancy versus 53.3% at 4 million per mL. These foundational studies collectively identified the three determinants of AI success that remain central to current protocol development: ovulation induction method, insemination timing relative to follicular status, and minimum effective sperm dose.

Subsequent research has revealed wide and persistent variability in AI pregnancy outcomes, ranging from 0% to approximately 73% depending on semen type, collection method, processing approach, and synchronization protocol (107, 108). Garcia and Alarcon (109) demonstrated in a field-scale study of 200 females that overall pregnancy rates of 50.5% are achievable with fresh semen diluted in Tris-glucose with egg yolk and deposited 26 to 30 h post-GnRH induction, rising to 73% with a second service to non-pregnant females. Garcia et al. (110) systematically evaluated the independent effects of follicle size, insemination timing, and sperm dose in 350 females, establishing that, pregnancy rate is not significantly affected by follicle diameter (7 to 9 mm vs. 10 to 12 mm) provided the 7 mm ovulatory threshold is exceeded; AI at 24 to 26 h post-induction achieves the highest pregnancy rates; and doses of 35 to 45 million spermatozoa per mL are substantially superior to 15 million per mL (50 to 55% vs. 30% pregnancy).

Post-coital vaginal aspiration yields ejaculates of lower viscosity and higher progressive motility than artificial vagina and offers field-level accessibility, but blood contamination from penile-induced epithelial abrasion compromises seminal evaluation accuracy, reduces representativeness of the aspirated sample, and introduces biosecurity concerns including the need for pathogen-verified stimulus females (13, 20, 111). Artificial vagina-based collection remains the preferred standard for research-grade semen acquisition and ART-focused processing. With respect to deposition route, transcervical deep intrauterine insemination and laparoscopic AI have yielded comparable results, with semen deposited closer to the oviduct consistently associated with higher fertility than pericervical deposition (77, 107). Both techniques require prior GnRH-confirmed ovulation induction and ultrasonographic identification of a dominant follicle of 7 mm or greater. Stuart et al. (101) demonstrated pregnancy following deep uterine AI with cryopreserved papain-treated spermatozoa, and Huamani et al. (103) reported current clinical benchmarks of 33.3 and 22.2% pregnancy with Biladyl B and AndroMed extenders, respectively, in frozen semen AI.

Despite this progress, AI remains largely confined to experimental settings. Key barriers to standardization include the absence of a validated minimum sperm dose for cooled and cryopreserved semen, incomplete definition of the optimal insemination interval across different ovulation induction agents, and persistent interindividual variability in semen quality and ovarian response that limits experimental reproducibility. Multi-site, adequately powered fertility trials designed around the operational framework established by Garcia and Alarcon (109) and Stuart et al. (101) are needed to establish standardized AI protocols for fresh, cooled, and cryopreserved alpaca semen under both experimental and commercial production conditions.

Artificial insemination protocols in dromedary camels are considerably more advanced than those currently available for alpacas, offering a practical benchmark for standardization efforts. Camel seminal plasma is routinely liquefied and diluted in extenders prior to intrauterine deposition, and pregnancy rates with fresh and liquid-stored semen at doses of 150 to 300 million motile spermatozoa are now sufficiently consistent to support commercial AI programs (92). The persistent AI barriers identified for alpacas in this review, an undefined minimum sperm dose for cooled and cryopreserved semen and variable insemination timing, have been more systematically addressed in camels through large, multi-year field trials directly comparing extender type, deposition site, and dose across hundreds of inseminations. Adapting this trial design framework to alpacas, rather than extrapolating dose thresholds from cattle or sheep AI, represents a direct path toward closing the standardization gap documented in this review.

### 4.2 Multiple ovulation and embryo transfer (MOET)

MOET offers the most direct pathway to rapid genetic improvement in alpacas by enabling genetically superior donors to produce multiple offspring per year, yet its application remains technically constrained and incompletely standardized. The first documented embryo transfer in alpacas was reported by Sumar and Franco (112), using 15 donors and 44 recipients; four pregnancies were established, yielding one live birth. The first report combining superovulation with non-surgical embryo transfer was published by Palomino et al. (113), who superovulated two donor alpacas to yield six and five transferable embryos respectively, transferred non-surgically into three synchronized recipients, and produced two full-term crias, establishing the proof of concept for MOET in this species.

The hormonal basis of superovulation has been most comprehensively investigated by Ratto et al. (63), who confirmed that pFSH administered in a decreasing-dose regimen of 100 to 200 mg over 2 to 4 days, or eCG at 700 to 1,000 IU as a single injection, both initiated 48 to 72 h after follicular wave emergence, consistently recruit 10 to 15 follicles per ovary under optimized conditions. More recently, Zampini et al. (7) demonstrated the feasibility of a simplified fixed-time GnRH-PGF2α-eCG synchronization and superstimulation protocol that does not require ultrasonographic follicular monitoring, an important practical advance for field-based MOET programs. Elimination of the dominant follicle prior to superstimulation initiation, whether by GnRH-induced ovulation or mechanical ablation, is now incorporated into all current protocol designs as a prerequisite for reliable cohort recruitment (31, 35).

Ovarian response to superovulatory treatment remains the most critical and least predictable variable in alpaca MOET, with reported ranges of 0 to more than 25 corpora lutea per donor in the same protocol and 20 to 30% of donors classified as non-responsive (19, 23). Embryo recovery by transcervical uterine flushing 7 to 9 days post-mating yields transferable embryos in 20 to 40% of corpora lutea, with recovery rate negatively correlated with total CL number, reflecting an inverse relationship between degree of superovulation and the quality of ovulatory and luteal events (19, 114). The anatomical predisposition of alpacas for embryo fixation in the left uterine horn, combined with the luteolytic asymmetry between uterine horns described in section 2.2, introduces additional complexity into recipient selection and embryo placement.

Large-scale retrospective analysis by Vaughan et al. (115) of 5,547 single and multiple ovulation embryo transfers on commercial Australian alpaca farms identified optimal flushing on days 8 to 9 post-mating, optimal transfer on days 7 to 8 post-GnRH, and recipient age above 15 years or body condition score below 2 as independent negative predictors of transfer success, providing the most statistically robust guidance currently available for commercial MOET program management.

Embryo cryopreservation represents the primary barrier to germplasm banking and international transport. Alpaca preimplantation embryos exhibit pronounced sensitivity to cryoinjury, with conventional slow-cooling protocols producing consistently low post-thaw survival rates. The critical breakthrough was achieved by Lutz et al. (116), who reported the first live cria birth following vitrified-warmed alpaca preimplantation embryo transfer using an open-pulled straw protocol incorporating sequential glycerol-ethylene glycol cryoprotectant loading. Despite this landmark, the number of live cria births from cryopreserved alpaca embryos reported in the peer-reviewed literature remains extremely low, and multi-site validation of vitrification protocols under research and commercial conditions constitutes a priority objective. Integration of MOET with genomic selection, including pre-transfer embryo sexing and genotyping by trophectoderm biopsy adapted from the cattle industry, represents the logical next step toward maximizing the genetic impact of each transfer campaign.

Embryo transfer in the dromedary camel has achieved a level of commercial maturity but has not yet been realized in alpacas, providing a template for scaling MOET programs. A large commercial camel embryo transfer program spanning two decades documented embryo recovery rates of 85% in single-ovulation cycles following superovulation, with standardized recipient synchronization and donor management protocols supporting sustained large-scale operation (117). The variability limiting alpaca MOET efficiency described above, 20 to 30% non-responder frequency and recovery rates negatively correlated with corpora lutea number, mirrors challenges documented and partially resolved in camel programs through recipient herd management, donor screening, and standardized flushing intervals. Direct application of these operational parameters, rather than continued reliance on bovine embryo transfer benchmarks, may accelerate the transition of alpaca MOET from an experimental to a commercially viable technology.

### 4.3 *In vitro* embryo production (IVEP)

IVEP requires a reliable source of COCs of sufficient developmental competence to support fertilization and embryo culture through the blastocyst stage. Two principal oocyte collection strategies have been evaluated: aspiration from slaughterhouse ovaries and OPU from live donors by ultrasound-guided transvaginal aspiration or laparoscopic approaches. Slaughterhouse-derived ovaries provide a logistically accessible and, cost-effective COC source for protocol development, consistently yielding higher mean COC numbers per ovary and a higher proportion of morphologically intact COCs than OPU from live animals (5, 15). However, the developmental competence of slaughterhouse-derived oocytes is systematically lower than that of oocytes from live superstimulated donors. Landeo et al. (5) demonstrated in a direct comparative study that OPU-derived oocytes, despite a higher proportion of morphologically inferior COCs, yielded significantly higher blastocyst rates per cleaved oocyte and per COC collected, confirming that the endocrine milieu of gonadotropin superstimulation confers a developmental advantage not replicated post-mortem.

LOPU, which allows direct visualization of the ovarian surface and targeted aspiration of follicles measuring 3 to 7 mm following synchronization and superstimulation, represents a significant practical advance in live-animal oocyte collection. In our laboratory, LOPU has consistently achieved recovery rates of up to 60% per aspirated follicle with an average of 6 to 8 COCs per ovary under FSH superstimulation (64), comparable to the 50 to 60% recovery rates and approximately 4.5 oocytes per ovary reported for ultrasound-guided transvaginal OPU. Concerns regarding progressive ovarian adhesions from repeated OPU sessions, documented in both llamas and alpacas following iterative ultrasound-guided procedures (14), underscore the importance of protocols that maximize oocyte yield per session and minimize cumulative ovarian trauma. COC selection based on morphological grade (grades I and II, defined by compact cumulus layers and homogeneous ooplasm) is a prerequisite for consistent IVEP outcomes (15, 18).

Brilliant cresyl blue (BCB) staining provides a metabolic complement to morphological grading. Castro-Modesto et al. (17) and Vargas-Donayre et al. (18) demonstrated that BCB-positive oocytes, which retain active glucose-6-phosphate dehydrogenase activity reflecting higher cytoplasmic maturation, exhibit significantly superior IVM and parthenogenetic activation outcomes than BCB-negative counterparts, suggesting that BCB selection may practically enrich the competent oocyte fraction, particularly in slaughterhouse-derived material where morphological grading is less reliable.

IVM is routinely performed in TCM-199 supplemented with gonadotropins, estradiol, and serum albumin at 38.5 °C, 5% CO_2_, and greater than 95% humidity for 24 to 32 h, with 32 h the most consistently reported duration associated with satisfactory metaphase II rates in slaughterhouse-derived material (15, 18). Ruiz et al. (15) demonstrated that 32-h maturation combined with low oxygen tension (5% O_2_) significantly improved cleavage and blastocyst rates relative to shorter maturation times or standard air oxygen tension (20% O_2_), establishing 5% O_2_ as the preferred atmospheric condition for alpaca *in vitro* embryo culture. *In vivo* maturation achieves superior nuclear maturation rates. Landeo et al. (5) reported *in vivo* MII rates approaching or exceeding 70% at 26 to 28 h following GnRH administration in superstimulated donors, which is substantially higher than conventional IVM of slaughterhouse COCs, and *in vivo*-matured oocytes subsequently exhibited higher blastocyst rates after IVF, confirming that current IVM systems do not fully replicate the developmental competence conferred by *in vivo* maturation.

IVF requires prior semen processing to eliminate viscous seminal plasma and enrich for motile, morphologically normal spermatozoa. Epididymal sperm processed by Percoll gradient centrifugation or swim-up are most commonly employed, given their more consistent quality relative to ejaculated semen (15, 99). Insemination is performed at 1 to 2 million motile spermatozoa per mL in modified TALP medium supplemented with heparin and a co-incubation period of 17 to 20 h at 38.5 °C (5). Presumptive zygotes are transferred to SOFaa supplemented with BSA or fetal bovine serum under low oxygen tension (15). Cleavage rates following IVF typically range from 30 to 60%, with blastocyst formation rates rarely exceeding 25% of cleaved embryos across published studies (5, 15, 19). Developmental arrest at the morula stage is frequently observed, attributed to intrinsic oocyte developmental deficiencies and the absence of fully species-adapted culture conditions. Critically, no live cria birth from conventional IVF with *in vitro*-cultured alpaca embryos has been reported in the peer-reviewed literature, in stark contrast to the live birth achieved by Lutz et al. (116) following vitrified embryo transfer from naturally mated non-superstimulated donors. Improvement of alpaca-specific embryo culture media, optimization of sequential media systems mirroring the oviductal and uterine biochemical environment, and systematic characterization of embryo metabolic requirements across developmental stages are the critical next steps toward achieving routine IVF-derived embryo transfer pregnancies.

#### 4.3.1 Intracytoplasmic sperm injection (ICSI)

ICSI bypasses the zona pellucida penetration and capacitation barriers that constrain conventional IVF, offering particular advantages when sperm availability is severely limited or when seminal plasma viscosity constitutes an insurmountable obstacle. The first published ICSI attempt in alpacas was by Castro-Modesto et al. (17), who achieved cleavage and morula formation following sperm injection and chemical activation, providing proof of concept for micromanipulation-based fertilization in this species.

A defining technical challenge of ICSI in alpacas is that sperm injection alone fails to trigger the calcium oscillation cascade required for full oocyte activation, necessitating artificial chemical activation protocols. Bellido-Quispe et al. (118) conducted the most systematic evaluation of activation protocols, comparing five treatments applied to oocytes matured for 30 h from slaughterhouse ovaries. The sequential ionomycin plus 6-DMAP protocol, incorporating a mandatory 3-h window period before 6-DMAP administration to allow second polar body extrusion and prevent artificial polyploidy induction, produced the highest cleavage and morula development rates, establishing it as the reference activation protocol for alpaca ICSI and aligning practice with the ionomycin-6-DMAP combination standard in bovine, ovine, and dromedary ICSI systems (118).

Despite these advances, ICSI in alpacas remains predominantly experimental. Blastocyst yields are consistently low, no pregnancies or live births from ICSI-derived alpaca embryos have been published, and the technique requires specialized micromanipulation equipment and controlled laboratory conditions not routinely available in South American production environments. Critical unresolved issues include the optimization of IVM duration and supplementation to maximize cytoplasmic maturation prior to injection, the development of sperm selection strategies for identifying functionally competent individual spermatozoa from the motility-impaired alpaca ejaculate, systematic comparison of piezo-drive versus conventional injection techniques, and the design of embryo culture systems capable of supporting development beyond the morula arrest stage consistently observed in current protocols (17, 19, 118).

*In vitro* embryo production outcomes in dromedary camels remain the most instructive comparator for alpaca IVF and ICSI, since both species share the same fundamental barriers of viscous, low-volume ejaculate and induced ovulation. The first fully *in vitro*-produced camelid offspring was a dromedary calf born after IVM, IVF with fresh semen in modified TALP medium, and culture in a semi-defined medium, demonstrating that the complete *in vitro* embryo production chain is achievable in Camelidae despite the low blastocyst rates characteristic of the family (119). Camel ICSI, first achieved using *in vitro*-matured oocytes injected with stored epididymal spermatozoa followed by ionomycin-roscovitine chemical activation, produced blastocyst-stage embryos through the same combined calcium-ionophore and kinase-inhibitor activation strategy later adopted for alpaca ICSI (120, 121). That no live offspring have yet been reported from ICSI in either camels or alpacas indicates that oocyte activation, rather than the injection procedure itself, remains the shared rate-limiting step across Camelidae, and that solutions developed in one species are likely to translate directly to the other.

## 5 Discussion

Alpaca reproductive biology operates through fundamentally distinct mechanistic processes compared to conventional livestock, creating both scientific opportunities and translational challenges that merit focused investigation. This review synthesizes evidence demonstrating that obligate induced ovulation mediated by seminal *β*-NGF, non-cyclic follicular wave dynamics, anatomical uterine asymmetry favoring left-horn implantation, and MUC5B-determined seminal plasma viscosity collectively establish a reproductive phenotype requiring species-tailored ART approaches. While significant progress has been achieved in understanding individual components of alpaca reproduction, integrated application of this knowledge toward developing field-viable, standardized protocols suitable for commercial genetic improvement programs remains incomplete.

The molecular characterization of *β*-NGF as the ovulation-inducing factor represents a watershed moment in camelid reproductive science, fundamentally reshaping our understanding of neurotrophin signaling in mammalian ovulation. Beyond triggering the preovulatory LH surge, *β*-NGF uniquely exerts luteotrophic effects through upregulation of StAR, CYP11A1, and 3*β*-HSD, distinguishing it mechanistically from conventional GnRH analogues and hCG (39, 51). Current pharmacological synchronization protocols fail to replicate these combined ovulatory and luteotrophic actions, suggesting that recombinant *β*-NGF preparations represent a rational next-generation approach for enhancing corpus luteum function and embryo survival in MOET and AI programs. This translational gap may substantially contribute to elevating embryonic loss rates currently limiting alpaca ART efficiency.

The identification and structural characterization of MUC5B as the principal viscosity determinant in alpaca seminal plasma has transformed semen cryopreservation from an intractable problem into a mechanistically addressable challenge. The sequential papain-E-64 liquefaction protocol achieved post-thaw motility values of 45 percent and confirmed pregnancies following frozen semen AI have established the proof-of-principle for cryopreservation in alpacas (100, 101). However, the requirement for approximately 10 percent residual seminal plasma to maintain sperm function reveals a fundamental paradox: viscosity reduction and sperm protection represent competing biological objectives that cannot be simultaneously optimized through current approaches. Future work should prioritize selective reconstitution of sperm-protective proteins, particularly phosphatidylethanolamine-binding protein 1 (PEBP1), which shows fertility-associated enrichment in low-viscosity samples and has been localized to alpaca oviductal fluid (95).

Ovarian response variability represents the primary inefficiency limiting current MOET programs, with reported follicle recruitment ranging from 0 to more than 25 per ovary within identical superstimulation protocols. While GnRH-induced dominant follicle elimination reliably triggers new wave emergence at 48 h, subsequent FSH/eCG responsiveness exhibits substantial interindividual variation unexplained by conventional management parameters (31, 63). Baseline antral follicle count assessment, a heritable trait predictive of reproductive capacity in cattle and sheep, has not been systematically evaluated in alpacas as a donor selection strategy. Integration of antral follicle count assessment-based donor stratification combined with genomic approaches identifying SNPs associated with ovarian responsiveness phenotypes would enable rational protocol customization and potentially reduce the 20 to 30 percent non-responder frequency currently compromising MOET efficiency.

The developmental competence gap distinguishing *in vivo* from *in vitro* embryo production in alpacas exceeds that documented in any other livestock species, with no peer-reviewed reports of live births from conventional IVF-derived embryos despite decades of protocol optimization efforts. *In vivo*-matured oocytes achieve metaphase II rates exceeding 70 percent, compared to substantially lower conventional IVM rates, indicating that current culture conditions fail to provide requisite biochemical signals for complete developmental competence acquisition (5, 15). The singular live cria birth from vitrified-warmed naturally-mated embryos (116) establishes that embryo cryopreservation is feasible, yet IVF-derived embryos consistently arrest at morula stage, implicating deficient *in vitro* oocyte competence rather than cryopreservation or transfer methodology as the limiting factor. Systematic metabolic profiling of preimplantation embryos coupled with transcriptomic analysis at developmental arrest points would identify species-specific nutrient requirements and signaling pathways currently unaddressed by standard culture media formulations.

Brilliant cresyl blue staining, which identifies oocytes retaining glucose-6-phosphate dehydrogenase activity and reflecting higher cytoplasmic maturation, demonstrated superior developmental outcomes compared to BCB-negative counterparts, suggesting metabolic biomarkers may enable practical enrichment for competent oocytes (17, 18). Integration of BCB selection with extended maturation duration and optimized supplementation represents an immediate advancement over current practices. Laparoscopic ovum pick-up has achieved recovery rates of 50 to 60 percent per aspirated follicle with average 6 to 8 oocytes per ovary under FSH superstimulation (64), demonstrating that live-animal oocyte collection is technically feasible at clinically applicable scale, though concerns regarding progressive ovarian adhesions from repeated procedures warrant investigation of protocols maximizing yield per session.

Artificial insemination pregnancy rates of 50 to 73 percent with fresh semen and 22 to 33 percent with cryopreserved semen, while demonstrating proof-of-concept, remain substantially below livestock benchmarks and exhibit unacceptable variability for commercial application (103, 109, 110). Current uncertainties regarding minimum effective sperm dose for cooled and cryopreserved semen, optimal insemination timing relative to GnRH administration, and mechanistic basis for dose–response relationships warrant multi-site fertility trials conducted under field conditions documenting environmental variables alongside genetic and individual responsiveness parameters. Such investigations would establish truly standardized protocols suitable for diverse alpaca production systems rather than current research-specific approaches with limited commercial applicability.

Advancing alpaca ART efficacy demands convergent research strategy integrating mechanistic reproductive biology with field-validated clinical trials. The alpaca represents growing global economic significance and harbors unique immunological traits essential to Andean heritage, positioning reproductive technology advancement as priority for sustainable genetic improvement. Coordinated multi-institutional investigation that bridges fundamental endocrinology, proteomics, embryology, and translational validation under production conditions remains necessary to realize the genetic improvement potential inherent in this species.

## 6 Conclusion

Alpaca reproduction is governed by a constellation of species-specific physiological traits that collectively distinguish it from conventional livestock and demand tailored, rather than borrowed, approaches to assisted reproduction. On the female side, obligate induced ovulation mediated by seminal *β*-NGF, continuous non-cyclic follicular wave dynamics with interwave intervals of 10 to 22 days, and a left-uterine-horn implantation bias exceeding 98% of pregnancies together define a reproductive rhythm with no direct equivalent in cattle or sheep. On the male side, mucin 5B-driven seminal plasma viscoelasticity, originating from bulbourethral gland secretions in the structural absence of seminal vesicles, imposes a further and largely independent constraint on semen handling. Three decades of targeted mechanistic investigation have generated proof-of-concept evidence that artificial insemination, multiple ovulation and embryo transfer, laparoscopic ovum pick-up, *in vitro* embryo production, and intracytoplasmic sperm injection are all biologically feasible in this species; none, however, has yet reached the standardization and field validation required for widespread commercial application.

The molecular identification of *β*-NGF as the ovulation-inducing factor stands as the most consequential advance underlying this progress. Acting systemically through kisspeptin-GnRH-LH activation, *β*-NGF confers luteotrophic support through upregulation of StAR, CYP11A1, and 3*β*-HSD in luteal tissue, a combined ovulatory and luteotrophic action that current GnRH analogue and hCG protocols fail to replicate. Translating this mechanistic understanding into a field-applicable recombinant *β*-NGF preparation is among the highest-priority objectives in the field, with direct implications for corpus luteum quality and embryonic loss across both AI and MOET programs. On the male side, sequential papain-E-64 liquefaction has moved semen cryopreservation from an intractable problem to a mechanistically addressable one, achieving post-thaw motility of up to 45% and confirmed pregnancies following frozen semen AI. Yet pregnancy rates of 22 to 33% with cryopreserved semen remain below livestock benchmarks, and the dual role of seminal plasma as both the principal source of viscosity and an indispensable sperm-protective medium constitutes the central unresolved paradox in this domain. Selective reconstitution of sperm-protective proteins, particularly PEBP1, alongside complete glycoform characterization of mucin 5B, represent the most tractable paths forward, while identification of the endogenous protease system responsible for spontaneous *in vivo* liquefaction remains a foundational open question whose resolution would enable biomimetic liquefaction strategies that eliminate viscosity without sacrificing sperm protection.

Among the assisted reproductive technologies reviewed, MOET has advanced furthest toward commercial reality, having been implemented at scale on Australian alpaca farms where GnRH-induced dominant follicle elimination reliably triggers new follicular wave emergence at 48 h and that optimized flushing at days 8 to 9 post-mating is now supported by large retrospective analyses. Its principal limitation is not technical feasibility but predictability: 20 to 30% of donors are classified as non-responders within identical superstimulation protocols, a variability that antral follicle count-based donor stratification combined with genomic identification of ovarian responsiveness phenotypes is best positioned to resolve, while the simplified fixed-time GnRH-PGF2α-eCG protocol requiring no ultrasonographic monitoring offers a practical route to broader field adoption. Laparoscopic ovum pick-up has likewise established live-animal oocyte collection at clinically applicable scale, with recovery rates of 50 to 60% per aspirated follicle and average yields of 6 to 8 COCs per ovary, though progressive ovarian adhesion formation following repeated sessions constitutes a procedural ceiling that will require systematic definition of safe session frequency thresholds to overcome.

*In vitro* embryo production remains the least mature of the technologies reviewed. The first live cria birth from a vitrified-warmed preimplantation embryo confirmed that cryopreservation is biologically feasible, yet this success followed natural mating rather than IVF, and conventional *in vitro*-fertilized embryos consistently arrest at the morula stage with no live birth yet reported in the peer-reviewed literature. The substantially higher developmental competence of *in vivo*-matured oocytes relative to their *in vitro*-matured counterparts identifies deficient cytoplasmic maturation, rather than fertilization or culture technique per se, as the primary bottleneck, pointing toward brilliant cresyl blue-based oocyte selection, reduced oxygen tension culture, and systematic metabolic and transcriptomic profiling of embryos at their developmental arrest points as the most direct means of closing this gap. Intracytoplasmic sperm injection with sequential ionomycin plus 6-DMAP activation has achieved cleavage and morula formation, establishing proof of concept for micromanipulation-based fertilization, but the absence of any reported pregnancy or live birth confirms that this technique remains firmly experimental, awaiting optimization of cytoplasmic maturation at the time of injection, selection strategies for identifying functionally competent spermatozoa from motility-impaired ejaculates, and culture systems capable of supporting development beyond the morula stage.

Closing the performance gap between these experimental outcomes and the reproductive efficiency long achieved in conventional livestock will require more than any single laboratory or technique in isolation. It will require coordinated, multi-institutional investigation that integrates reproductive endocrinology, seminal plasma proteomics, embryo developmental biology, and large-scale, adequately powered field fertility trials capable of establishing standardized minimum sperm doses, insemination intervals, and extender formulations under real production conditions. The alpaca’s distinctive immunological traits and its enduring significance to Andean cultural heritage make this convergence not merely a scientific opportunity but a practical necessity, positioning coordinated advances in reproductive technology as a priority for sustainable, globally applicable genetic improvement in this species.

## References

[1] Kadwell M Fernandez M Stanley HF Baldi R Wheeler JC Rosadio R . Genetic analysis reveals the wild ancestors of the llama and the alpaca. Proc R Soc Lond Ser B Biol Sci. (2001) 268:2575–84. doi: 10.1098/rspb.2001.1774, 11749713 PMC1088918

[2] Quispe EC Rodríguez TC Iñiguez LR Mueller JP. Producción De Fibra De Alpaca, Llama, Vicuña Y Guanaco En Sudamérica. Anim Genet Resour Inf. (2009) 45:1–14. doi: 10.1017/S1014233909990277

[3] Hamers-Casterman C Atarhouch T Muyldermans S Robinson G Hamers C Songa EB . Naturally occurring antibodies devoid of light chains. Nature. (1993) 363:446–8. doi: 10.1038/363446a0 8502296

[4] Morrell JM Abraham MC. Semen handling in south American camelids: state of the art. Front Vet Sci. (2020) 7:2020. doi: 10.3389/fvets.2020.586858 PMC7677228 33240960

[5] Landeo L Zuñiga M Gastelu T Artica M Ruiz J Silva M . Oocyte quality, in vitro fertilization and embryo development of alpaca oocytes collected by ultrasound-guided follicular aspiration or from slaughterhouse ovaries. Animals. (2022) 12:1102. doi: 10.3390/ani12091102, 35565530 PMC9102040

[6] Bravo WP. Chapter 27 - artificial insemination of llamas and alpacas. In: Cebra C Anderson DE Tibary A Saun RJ Van Johnson LW, editors. Llama and Alpaca Care. St. Louis: W.B. Saunders (2014). pp. 310–315.

[7] Zampini EG Veiga MF Fumuso FG Cabido L Neild DM Chaves MG . Development of a Gnrh-Pgf2α based synchronization and superstimulation protocol for fixed-time mating in llama embryo donors. Front Vet Sci. (2020) 7:2020. doi: 10.3389/fvets.2020.595889 PMC7688663 33282933

[8] Fernández-Baca S. "Alpaca raising in the high Andes". In: Animal Breeding: Selected Articles from the World Animal Review. Rome, Italy: Food and Agriculture Organization of the United Nations (1993)

[9] Ratto MH Huanca W Singh J Adams GP. Local versus systemic effect of ovulation-inducing factor in the seminal plasma of alpacas. Reprod Biol Endocrinol. (2005) 3:29. doi: 10.1186/1477-7827-3-29, 16018817 PMC1190216

[10] Vaughan J. Ovarian function in south American camelids (alpacas, llamas, vicunas, guanacos). Anim Reprod Sci. (2011) 124:237–43. doi: 10.1016/j.anireprosci.2010.08.031, 20875933

[11] Adams GP Sumar J Ginther OJ. Effects of lactational and reproductive status on ovarian follicular waves in llamas (Lama glama). Reprod. (1990) 90:535–45. doi: 10.1530/jrf.0.0900535, 2250251

[12] Kershaw-Young CM Stuart C Evans G Maxwell WMC. The effect of glycosaminoglycan enzymes and proteases on the viscosity of alpaca seminal plasma and sperm function. Anim Reprod Sci. (2013) 138:261–7. doi: 10.1016/j.anireprosci.2013.02.005, 23537479

[13] Bravo PW Ccallo M Garnica J. The effect of enzymes on semen viscosity in llamas and alpacas. Small Rumin Res. (2000) 38:91–5. doi: 10.1016/s0921-4488(00)00142-5, 10924884

[14] Tibary A Ruiz A. "Laparoscopia Y Cirugías Reproductivas Laparoscópicas En Llamas Y Alpacas". In: Mellisho E, editor. Técnicas De Investigación Reproductiva En Camélidos. Peru: Asociación Peruana de Reproducción Animal (ASPRA) (2018). p. 55–66.

[15] Ruiz J Paulo Santayana R José Mendoza M Leandra Landeo J Huamán E Ticllacuri F . Effect of oocyte maturation time, sperm selection method and oxygen tension on in vitro embryo development in alpacas. Theriogenology. (2017) 95:127–32. doi: 10.1016/j.theriogenology.2017.03.006, 28460666

[16] Paiva L Silva M Carrasco R Ratto MH. The ovulatory and luteotropic actions of the male-derived Beta-nerve growth factor in south American camelids. Anim Front. (2022) 12:87–94. doi: 10.1093/af/vfac037, 35974784 PMC9374510

[17] Castro-Modesto T Mamani P Pella R Bravo Z Villarreal-Ugarte S Cancho C . Effect of follicle size on in vitro maturation in alpaca oocytes (Vicugna pacos) and the first ICSI in alpaca species. Small Rumin Res. (2022) 213:106680. doi: 10.1016/j.smallrumres.2022.106680

[18] Vargas-Donayre D Bravo Z Zeña H Quispe J Levano G Ratto M . Effect of brilliant cresyl blue (BCB) staining in oocyte’s maturation and parthenogenetic activation in alpaca (Vicugna pacos) oocytes. Small Rumin Res. (2024) 231:107175. doi: 10.1016/j.smallrumres.2023.107175

[19] Trasorras VL Carretero MI Neild DM Chaves MG Giuliano SM Miragaya MH. Production, preservation, and transfer of south American camelid embryos. Front Vet Sci. (2017) 4:190. doi: 10.3389/fvets.2017.00190, 29181380 PMC5693846

[20] Carretero MI Giuliano SM Miragaya MH Neild DM. Male reproductive biotechnologies in south American camelids part I: semen collection, evaluation and handling. Anim Reprod Sci. (2025) 272:107634. doi: 10.1016/j.anireprosci.2024.107634 39541760

[21] Sumar J Adams GP. "Reproductive anatomy and life cycle of the male and female llama and alpaca". In: Youngquist RS Threlfall WR, editors. Current Therapy in Large Animal Theriogenology, 2nd Edn. St. Louis: Saunders Elsevier (2009). p. 856–72.

[22] Aba MA. Chapter 14 - anatomy and physiology of reproduction in the female llama and alpaca. In: Cebra C Anderson DE Tibary A Saun RJ Van Johnson LW, editors. Llama and Alpaca Care. St. Louis: W.B. Saunders (2014). pp. 140–150

[23] Tibary A Anouassi A. Theriogenology in Camelidae: Anatomy, Physiology, Pathology and Artificial Breeding. Abu Dhabi: Abu Dhabi Printing and Publishing Company (1997).

[24] Arroyo E Patiño C Ciccarelli M Raudsepp T Conley A Tibary A. Clinical and histological features of ovarian hypoplasia/dysgenesis in alpacas. Front Vet Sci. (2022) 9:837684. doi: 10.3389/fvets.2022.837684, 35400100 PMC8990812

[25] Brennan PLR Purdy S Bacon SJ. Intra-horn insemination in the alpaca Vicugna Pacos: copulatory wounding and deep sperm deposition. PLoS One. (2024) 19:e0295882. doi: 10.1371/journal.pone.0295882, 38630763 PMC11023217

[26] Brennan PLR Sterett M DiBuono M Lara Granados G Klo K Marsden R . Intra-horn penile intromission in the alpaca Vicugna pacos and consequences to genital morphology. Integr Comp Biol. (2021) 61:624–33. doi: 10.1093/icb/icab050, 33970265

[27] Picha Y Tibary A Memon M Kasimanickam R Sumar J. Chronology of early embryonic development and embryo uterine migration in alpacas. Theriogenology. (2013) 79:702–8. doi: 10.1016/j.theriogenology.2012.11.027, 23290313

[28] Argañaraz ME Apichela SA Zampini R Vencato J Stelletta C. Biochemical and protein profile of alpaca (Vicugna pacos) uterine horn fluid during early pregnancy. Reprod Domest Anim. (2015) 50:121–8. doi: 10.1111/rda.12460, 25472782

[29] Sánchez-Caycho K Chávez-Quispe R Mellisho E. Histomorphometric comparison of left and right oviduct structure from alpaca (Vicugna pacos). Anat Histol Embryol. (2023) 52:673–83. doi: 10.1111/ahe.12924 37138523

[30] San-Martin M Copaira M Zuniga J Rodreguez R Bustinza G Acosta L. Aspects of reproduction in the alpaca. Reproduction. (1968) 16:395–9. doi: 10.1530/jrf.0.0160395, 5673739

[31] Adams GP Ratto MH Carrasco RA. Natural and controlled ovulation in south American camelids. Anim Reprod. (2018) 15:996–1002. doi: 10.21451/1984-3143-ar2018-0033, 36249856 PMC9536045

[32] Vaughan JL Macmillan KL D’Occhio MJ. Ovarian follicular wave characteristics in alpacas. Anim Reprod Sci. (2004) 80:353–61. doi: 10.1016/j.anireprosci.2003.08.002, 15036510

[33] Aba MA. "Anatomy and physiology of reproduction in the female llama and alpaca". In: Youngquist RS Threlfall WR, editors. Current Therapy in Large Animal Theriogenology, 2nd Edn. St. Louis: Saunders Elsevier (2007). p. 877–84.

[34] Bravo PW Stabenfeldt GH Lasley BL Fowler ME. The effect of ovarian follicle size on pituitary and ovarian responses to copulation in domesticated south American camelids. Biol Reprod. (1991) 45:553–9. doi: 10.1095/biolreprod45.4.553, 1751629

[35] Gallelli MF Bianchi C Trasorras V Zampini E Aba M Miragaya M. Synchronization of time of development of ovarian follicular waves in south American camelids. Anim Reprod Sci. (2019) 208:106105. doi: 10.1016/j.anireprosci.2019.06.017, 31405457

[36] Sumar JB. Reproduction in female south American domestic camelids. J Reprod Fertil Suppl. (1999) 54:169–78. doi: 10.1530/biosciprocs.4.013, 10692853

[37] Bravo PW Fowler ME Stabenfeldt GH Lasley B. Endocrine responses in the llama to copulation. Theriogenology. (1990) 33:891–9. doi: 10.1016/0093-691x(90)90824-d 16726785

[38] Campbell AJ Pearson LK Spencer TE Tibary A. Double ovulation and occurrence of twinning in alpacas (Vicugna pacos). Theriogenology. (2015) 84:421–4. doi: 10.1016/j.theriogenology.2015.03.027 25963129

[39] Ratto MH Leduc YA Valderrama XP van Straaten KE Delbaere LT Pierson RA . The nerve of ovulation-inducing factor in semen. Proc Natl Acad Sci USA. (2012) 109:15042–7. doi: 10.1073/pnas.1206273109 22908303 PMC3443178

[40] Sumar J Fredriksson G Alarcón V Kindahl H Edqvist LE. Levels of 15-Keto-13,14-dihydro-Pfg2 alpha, progesterone and Oestradiol-17 Beta after induced ovulations in llamas and alpacas. Acta Vet Scand. (1988) 29:339–46. doi: 10.1186/bf03548627, 3256233 PMC8161624

[41] Paolicchi F Urquieta B Del Valle L Bustos-Obregón E. Biological activity of the seminal plasma of alpacas: stimulus for the production of Lh by pituitary cells. Anim Reprod Sci. (1999) 54:203–10. doi: 10.1016/s0378-4320(98)00150-x, 10066107

[42] Adams GP Ratto MH Huanca W Singh J. Ovulation-inducing factor in the seminal plasma of alpacas and Llamas1. Biol Reprod. (2005) 73:452–7. doi: 10.1095/biolreprod.105.040097, 15888733

[43] Kershaw-Young CM Evans G Maxwell WM. Glycosaminoglycans in the accessory sex glands, testes and seminal plasma of alpaca and ram. Reprod Fertil Dev. (2012) 24:362–9. doi: 10.1071/rd11152, 22281083

[44] Adams GP Ratto MH Silva ME Carrasco RA. Ovulation-inducing factor (Oif/Ngf) in seminal plasma: a review and update. Reprod Domest Anim. (2016) 51:4–17. doi: 10.1111/rda.12795, 27762054

[45] Bogle OA Ratto MH Adams GP. Ovulation-inducing factor (Oif) induces Lh secretion from pituitary cells. Anim Reprod Sci. (2012) 133:117–22. doi: 10.1016/j.anireprosci.2012.06.006, 22770553

[46] El Allali K El Bousmaki N Ainani H Simonneaux V. Effect of the camelid's seminal plasma ovulation-inducing factor/*β*-Ngf: a kisspeptin target hypothesis. Front Vet Sci. (2017) 4:99. doi: 10.3389/fvets.2017.00099, 28713816 PMC5491598

[47] Berland M Paiva L Santander LA Ratto MH. Distribution of Gnrh and kisspeptin immunoreactivity in the female llama hypothalamus. Front Vet Sci. (2020) 7:597921. doi: 10.3389/fvets.2020.597921, 33604362 PMC7884347

[48] Silva ME Smulders JP Guerra M Valderrama XP Letelier C Adams GP . Cetrorelix suppresses the preovulatory Lh surge and ovulation induced by ovulation-inducing factor (Oif) present in llama seminal plasma. Reprod Biol Endocrinol. (2011) 9:74. doi: 10.1186/1477-7827-9-74, 21624125 PMC3123631

[49] Ulloa-Leal C Bogle OA Adams GP Ratto MH. Luteotrophic effect of ovulation-inducing factor/nerve growth factor present in the seminal plasma of llamas. Theriogenology. (2014) 81:1101–7.e1. doi: 10.1016/j.theriogenology.2014.01.038, 24582374

[50] Silva M Ulloa-Leal C Norambuena C Fernández A Adams GP Ratto MH. Ovulation-inducing factor (Oif/Ngf) from seminal plasma origin enhances Corpus luteum function in llamas regardless the preovulatory follicle diameter. Anim Reprod Sci. (2014) 148:221–7. doi: 10.1016/j.anireprosci.2014.05.012, 24950997

[51] Silva M Ulloa-Leal C Valderrama XP Bogle OA Adams GP Ratto MH. Nerve growth factor from seminal plasma origin (Sp*β*-Ngf) increases cl vascularization and level of mRNA expression of steroidogenic enzymes during the early stage of corpus luteum development in llamas. Theriogenology. (2017) 103:69–75. doi: 10.1016/j.theriogenology.2017.07.041 28779611

[52] Carrasco R Singh J Adams GP. The dynamics of Trka expression in the bovine ovary are associated with a Luteotrophic effect of ovulation-inducing factor/nerve growth factor (Oif/Ngf). Reprod Biol Endocrinol. (2016) 14:47. doi: 10.1186/s12958-016-0182-9, 27542717 PMC4992250

[53] Bogle OA Carrasco RA Ratto MH Singh J Adams GP. Source and localization of ovulation-inducing factor/nerve growth factor in male reproductive tissues among mammalian species. Biol Reprod. (2018) 99:1194–204. doi: 10.1093/biolre/ioy149, 29982342

[54] Chen BX Yuen ZX Pan GW. Semen-induced ovulation in the Bactrian camel (Camelus Bactrianus). J Reprod Fertil. (1985) 74:335–9. doi: 10.1530/jrf.0.0740335, 3900379

[55] Ratto MH Huanca W Singh J Adams GP. Comparison of the effect of ovulation-inducing factor (Oif) in the seminal plasma of llamas, alpacas, and bulls. Theriogenology. (2006) 66:1102–6. doi: 10.1016/j.theriogenology.2006.02.050, 16630652

[56] Aba MA Kindahl H Forsberg M Quiroga M Auza N. Levels of progesterone and changes in prostaglandin F(2alpha) release during Luteolysis and early pregnancy in llamas and the effect of treatment with flunixin meglumine. Anim Reprod Sci. (2000) 59:87–97. doi: 10.1016/s0378-4320(00)00068-3, 10804278

[57] Fernandez-Baca S Hansel W Novoa C. Corpus luteum function in the alpaca12. Biol Reprod. (1970) 3:252–61. doi: 10.1093/biolreprod/3.2.252 5522857

[58] Bianchi CP Gallelli MF Herrera JM Benavente MA Rossetto L Aba MA. Current knowledge about the processes of Luteolysis and maternal recognition of pregnancy in camelids. Reprod Domest Anim. (2023) 58:3–9. doi: 10.1111/rda.14269, 36149369

[59] Del Campo MR Del Campo CH Adams GP Mapletoft RJ. The application of new reproductive technologies to south American camelids. Theriogenology. (1995) 43:21–30. doi: 10.1016/0093-691X(94)00007-H

[60] Fernandez-Baca S Hansel W Saatman R Sumar J Novoa C. Differential luteolytic effects of right and left uterine horns in the alpaca. Biol Reprod. (1979) 20:586–95. doi: 10.1095/biolreprod20.3.586, 572239

[61] Tibary A Anouassi A Sghiri A Khatir H. Current knowledge and future challenges in camelid reproduction. Soc Reprod Fertil Suppl. (2007) 64:297–313. doi: 10.5661/rdr-vi-297, 17491155

[62] Vaughan J. Glove Box Guide to Alpacas. Mitcham: Australian Alpaca Association (2018).

[63] Ratto MH Silva ME Huanca W Huanca T Adams GP. Induction of superovulation in south American camelids. Anim Reprod Sci. (2013) 136:164–9. doi: 10.1016/j.anireprosci.2012.10.006, 23146202

[64] Landinez-Aponte J Joo M Stott R Merrill N Cisneros J Liu Y . Laparoscopic ovum pick-up for in vivo oocyte retrieval in alpacas. J Vis Exp (2026). 232:e71397. doi: 10.3791/71397 42371936

[65] Tibary A Vaughan J. Reproductive physiology and infertility in male south American camelids: a review and clinical observations. Small Rumin Res. (2006) 61:283–98. doi: 10.1016/j.smallrumres.2005.07.018

[66] Bravo PW Johnson LW. Reproductive physiology of the male camelid. Vet Clin North Am Food Anim Pract. (1994) 10:259–64. doi: 10.1016/s0749-0720(15)30560-0, 7953959

[67] Pérez-Durand MG Massa-Guzmán A Luque-Mamani N Ruelas-Calloapaza DA Urviola-Sánchez JM Condori-Chuchi EA . Age-related differences in testosterone concentration and its relation to testicular biometrics, hemodynamics, and fertility in alpacas (Vicugna pacos). Vet Sci. (2023) 10:429. doi: 10.3390/vetsci10070429, 37505834 PMC10385440

[68] Liguori G Paino S Squillacioti C Luca D Alì S Langella E . Innervation and immunohistochemical characteristics of epididymis in alpaca camelid (Vicugna pacos). Ital J Anim Sci. (2013) 12:e15. doi: 10.4081/ijas.2013.e15

[69] Kershaw-Young C Maxwell W. Seminal plasma components in camelids and comparisons with other species. Reprod Domest Anim. (2012) 47:369–75. doi: 10.1111/j.1439-0531.2012.02100.x, 22827394

[70] Adams GP Ratto MH. Ovulation-inducing factor in seminal plasma: a review. Anim Reprod Sci. (2013) 136:148–56. doi: 10.1016/j.anireprosci.2012.10.004, 23141951

[71] El Zawam A Tibary A Patino C. Basal levels and Hcg responses of serum testosterone and estrogen in male alpacas. Front Vet Sci. (2020) 7:595856. doi: 10.3389/fvets.2020.595856 33263018 PMC7688520

[72] Valdivia M Bravo Z Reyes J Gonzales GF. Rescue and conservation of male adult alpacas (Vicugna pacos) based on spermatogonial stem cell biotechnology using atomized black maca as a supplement of cryopreservation medium. Front Vet Sci. (2021) 8:597964. doi: 10.3389/fvets.2021.597964 33816583 PMC8010694

[73] Stelletta C Juyena NS Ponce Salazar D Ruiz J Gutierrez G. Testicular cytology of alpaca: comparison between impressed and smeared slides. Anim Reprod Sci. (2011) 125:133–7. doi: 10.1016/j.anireprosci.2011.03.005 21493020

[74] Wang H Dong Y Chen W Hei J Dong C. Expression and localization of nerve growth factor (Ngf) in the testis of alpaca (llama pacos). Folia Histochem Cytobiol. (2011) 49:55–61. doi: 10.5603/fhc.2011.0009 21526490

[75] Sari LM Zampini R Argañaraz ME Carretero MI Fumuso FG Barraza DE . Expression of *β*-Ngf and high-affinity Ngf receptor (Trka) in llama (Lama glama) male reproductive tract and spermatozoa. Mol Reprod Dev. (2018) 85:934–44. doi: 10.1002/mrd.23075 30328213

[76] Mann T. The Biochemistry of Semen and of the Male Reproductive Tract. 2d ed. London: Methuen (1964).

[77] Bravo PW Flores U Garnica J Ordoñez C. Collection of semen and artificial insemination of alpacas. Theriogenology. (1997) 47:619–26. doi: 10.1016/s0093-691x(97)00020-4, 16728014

[78] Garnica J Achata R Bravo PW. Physical and biochemical characteristics of alpaca semen. Anim Reprod Sci. (1993) 32:85–90. doi: 10.1016/0378-4320(93)90059-Z

[79] Garnica J Flores E Bravo W. Citric acid and fructose concentrations in seminal plasma of the alpaca. Small Rumin Res. (1995) 18:95–8. doi: 10.1016/0921-4488(95)00720-6

[80] Druart X Rickard JP Mactier S Kohnke PL Kershaw-Young CM Bathgate R . Proteomic characterization and cross species comparison of mammalian seminal plasma. J Proteome. (2013) 91:13–22. doi: 10.1016/j.jprot.2013.05.029, 23748023

[81] Ali HA Moniem KA Tingari MD. Some histochemical studies on the prostate, urethral and bulbourethral glands of the one-humped camel (Camelus Dromedarius). Histochem J. (1976) 8:565–78. doi: 10.1007/bf01003958, 186443

[82] Perk K. Seasonal changes in the glandula bulbo-urethralis of the camel. Bull Res Counc Isr Sect E Exp Med. (1962) 10:37–44. 14038281

[83] Aisen EG Huanca López W Pérez Durand MG Torres Mamani E Villanueva Mori JC Ousset MJ . Spermatozoa obtained from alpaca vas deferens. Effects of seminal plasma added at post-thawing. Front Vet Sci. (2021) 8:611301. doi: 10.3389/fvets.2021.611301, 33644145 PMC7902725

[84] Thérien I Bergeron A Bousquet D Manjunath P. Isolation and characterization of glycosaminoglycans from bovine follicular fluid and their effect on sperm capacitation. Mol Reprod Dev. (2005) 71:97–106. doi: 10.1002/mrd.20287, 15736127

[85] Bergqvist AS Ballester J Johannisson A Hernandez M Lundeheim N Rodríguez-Martínez H. In vitro capacitation of bull spermatozoa by oviductal fluid and its components. Zygote. (2006) 14:259–73. doi: 10.1017/s0967199406003777, 16822337

[86] Mogielnicka-Brzozowska M Kordan W. Characteristics of selected seminal plasma proteins and their application in the improvement of the reproductive processes in mammals. Pol J Vet Sci. (2011) 14:489–99. doi: 10.2478/v10181-011-0074-z 21957748

[87] Kesimer M Sheehan JK. Mass spectrometric analysis of mucin Core proteins. Methods Mol Biol. (2012) 842:67–79. doi: 10.1007/978-1-61779-513-8_4, 22259130 PMC5151179

[88] Russo CL Spurr-Michaud S Tisdale A Pudney J Anderson D Gipson IK. Mucin gene expression in human male urogenital tract epithelia. Hum Reprod. (2006) 21:2783–93. doi: 10.1093/humrep/del164 16997931 PMC2893033

[89] Boursnell JC Hartree EF Briggs PA. Studies of the Bulbo-urethral (Cowper's)-gland mucin and seminal gel of the boar. Biochem J. (1970) 117:981–8. doi: 10.1042/bj1170981, 5451918 PMC1179059

[90] Kesimer M Sheehan JK. Analyzing the functions of large glycoconjugates through the dissipative properties of their absorbed layers using the gel-forming mucin Muc5b as an example. Glycobiology. (2008) 18:463–72. doi: 10.1093/glycob/cwn024 18339669

[91] Kershaw-Young CM Maxwell WM. The effect of seminal plasma on alpaca sperm function. Theriogenology. (2011) 76:1197–206. doi: 10.1016/j.theriogenology.2011.05.016 21820722

[92] Skidmore JA Malo CM Crichton EG Morrell JM Pukazhenthi BS. An update on semen collection, preservation and artificial insemination in the dromedary camel (Camelus dromedarius). Anim Reprod Sci. (2018) 194:11–8. doi: 10.1016/j.anireprosci.2018.03.013 29572045

[93] Mal G Vyas S Srinivasan A Patil NV Pathak KM. Studies on liquefaction time and proteins involved in the improvement of seminal characteristics in dromedary camels (Camelus dromedarius). Scientifica. (2016) 2016:4659358. doi: 10.1155/2016/4659358 27022505 PMC4775800

[94] D'Amours O Frenette G Fortier M Leclerc P Sullivan R. Proteomic comparison of detergent-extracted sperm proteins from bulls with different fertility indexes. Reproduction. (2010) 139:545–56. doi: 10.1530/rep-09-0375 19952166

[95] Torres Hualla EA Martiarena A Buglio Ballesteros MG Medina Rojas MF Rivera Chino C Gandarillas Espezua D . Proteomic and functional analysis of alpaca (Vicugna pacos) sperm quality following in vitro capacitation with follicular and oviductal fluids. Front Vet Sci. (2025) 12:1702095. doi: 10.3389/fvets.2025.1702095 41280419 PMC12631973

[96] Santiani A Huanca W Sapana R Huanca T Sepúlveda N Sánchez R. Effects on the quality of frozen-thawed alpaca (Lama Pacos) semen using two different cryoprotectants and extenders. Asian J Androl. (2005) 7:303–9. doi: 10.1111/j.1745-7262.2005.00021.x, 16110359

[97] Morton KM Bathgate R Evans G Maxwell WM. Cryopreservation of epididymal alpaca (Vicugna Pacos) sperm: a comparison of citrate-, tris- and lactose-based diluents and pellets and straws. Reprod Fertil Dev. (2007) 19:792–6. doi: 10.1071/rd07049, 17897581

[98] Morton KM Evans G Maxwell WM. Effect of glycerol concentration, Equex Stm supplementation and liquid storage prior to freezing on the motility and acrosome integrity of frozen-thawed epididymal alpaca (Vicugna pacos) sperm. Theriogenology. (2010) 74:311–6. doi: 10.1016/j.theriogenology.2010.02.015 20416935

[99] Mamani-Mango G Moina Gonzales M Ramos Hidalgo M Mendoza Mallma J Ruiz Béjar J Rivas Palma V . Effect of extender and freezing rate on quality parameters and in vitro fertilization capacity of alpaca spermatozoa recovered from cauda epididymis. Biopreserv Biobank. (2019) 17:39–45. doi: 10.1089/bio.2018.0021 30256664

[100] Kershaw CM Evans G Rodney R Maxwell WMC. Papain and its inhibitor E-64 reduce camelid semen viscosity without impairing sperm function and improve post-thaw motility rates. Reprod Fertil Dev. (2017) 29:1107–14. doi: 10.1071/rd15261, 27156102

[101] Stuart CC Vaughan JL Kershaw CM de Graaf SP Bathgate R. Effect of diluent type, cryoprotectant concentration, storage method and freeze/thaw rates on the post-thaw quality and fertility of cryopreserved alpaca spermatozoa. Sci Rep. (2019) 9:12826. doi: 10.1038/s41598-019-49203-z, 31492923 PMC6731240

[102] Levano G Quispe J Vargas D García M López A Aguila L . Effect of atomized black maca (Lepidium meyenii) supplementation in the cryopreservation of alpaca (Vicugna pacos) epididymal spermatozoa. Animals. (2023) 13:2054. doi: 10.3390/ani13132054, 37443852 PMC10339992

[103] Huamani MC Olaguivel Flores CA Palomino CYG Carretero MI Fumuso FG Arcce IML. Pregnancy and birth rate outcomes in alpacas (Vicugna pacos) inseminated with frozen semen using two commercial extenders. Reprod Domest Anim. (2024) 59:e14514. doi: 10.1111/rda.14514 38054582

[104] Fernandez-Baca S Novoa C. Primer Ensayo De Inseminacion Artificial De Alpacas (Lama-Pacos) Con Semen De Vicuña (Vicugna-Vicugna). Rev Fac Med Vet. (1968) 22:9–18.

[105] Calderon W Sumar L Franco E. Avances En La Inseminacion Artificial De Las Alpacas (Lama Pacos). Rev Fac Med Vet. (1968) 22:19–35.

[106] Bravo PW Pacheco C Quispe G Vilcapaza L Ordoñez C. Degelification of alpaca semen and the effect of dilution rates on artificial insemination outcome. Arch Androl. (1999) 43:239–46. doi: 10.1080/014850199262562, 10624509

[107] Bravo PW Alarcon V Baca L Cuba Y Ordoñez C Salinas J . Semen preservation and artificial insemination in domesticated south American camelids. Anim Reprod Sci. (2013) 136:157–63. doi: 10.1016/j.anireprosci.2012.10.005, 23153624

[108] Al-Essawe EM Abraham C Kunkitti P Axnér E de Verdier K Båge R . Extenders for alpaca epididymal spermatozoa: comparison of Inra96 and Andromed. Anim Reprod Sci. (2020) 223:106629. doi: 10.1016/j.anireprosci.2020.106629, 33126045

[109] García VW Alarcón BV. Efecto De La Inseminación Artificial Con Semen Fresco, Con Uno Y Dos Servicios, En La Fertilidad Y Natalidad De Las Alpacas. Rev Investig Vet Peru. (2019) 30:760–7. doi: 10.15381/rivep.v30i2.16072

[110] Garcia VW Pacheco Curie J Catacora Flores N Cardenas Suares N. Factores Que Influyen En La Inseminación Artificial Con Semen Fresco En Alpacas (Vicugna Pacos). Rev Investig Vet Peru. (2024) 35:e25890. doi: 10.15381/rivep.v35i5.25890

[111] Alarcón VB García VW Bravo PW. Inseminación Artificial De Alpacas Con Semen Colectado Por Aspiración Vaginal Y Vagina Artificial. Rev Investig Vet Peru. (2012) 23:58–64. doi: 10.15381/rivep.v23i1.882

[112] Sumar J Franco E Ensayos De Transferencia De Embriones En Alpacas In Informe Final Ivita-La Raya Lima, Perú: Universidad Nacional Mayor de San Marcos (1974)

[113] Palomino M Tabacchi L Avila E Eli O. Ensayo Preliminar De Transferencia De Embriones En Camélidos Sudamericanos. Revista de Camélidos Sudamericanos. (1987) 5:10–7.

[114] Correa JE Ratto MH Gatica R. Superovulation in llamas (Lama Glama) with Pfsh and equine chorionic gonadotrophin used individually or in combination. Anim Reprod Sci. (1997) 46:289–96. doi: 10.1016/s0378-4320(96)01615-6, 9231267

[115] Vaughan J Mihm M Wittek T. Factors influencing embryo transfer success in alpacas—a retrospective study. Anim Reprod Sci. (2013) 136:194–204. doi: 10.1016/j.anireprosci.2012.10.010, 23141430

[116] Lutz JC Johnson SL Duprey KJ Taylor PJ Vivanco-Mackie HW Ponce-Salazar D . Birth of a live cria after transfer of a vitrified-warmed alpaca (Vicugna pacos) preimplantation embryo. Front Vet Sci. (2020) 7:581877. doi: 10.3389/fvets.2020.581877 33344527 PMC7744456

[117] Anouassi A Tibary A. Development of a large commercial camel embryo transfer program: 20 years of scientific research. Anim Reprod Sci. (2013) 136:211–21. doi: 10.1016/j.anireprosci.2012.10.012, 23153626

[118] Bellido-Quispe DK Mujica Lengua FR Contreras Huamani M Palomino JM. Effect of chemical activators after intracytoplasmic sperm injection (Icsi) on embryo development in alpacas. Anim Reprod Sci. (2024) 263:107432. doi: 10.1016/j.anireprosci.2024.107432, 38401395

[119] Khatir H Anouassi A. The first dromedary (Camelus Dromedarius) offspring obtained from in vitro matured, in vitro fertilized and in vitro cultured abattoir-derived oocytes. Theriogenology. (2006) 65:1727–36. doi: 10.1016/j.theriogenology.2005.09.029, 16263162

[120] Wani NA. In vitro embryo production (Ivep) in camelids: present status and future perspectives. Reprod Biol. (2021) 21:100471. doi: 10.1016/j.repbio.2020.100471, 33307379

[121] Wani NA Hong S. Intracytoplasmic sperm injection (Icsi) of in vitro matured oocytes with stored epididymal spermatozoa in camel (Camelus Dromedarius): effect of exogenous activation on in vitro embryo development. Theriogenology. (2018) 113:44–9. doi: 10.1016/j.theriogenology.2018.02.004, 29454297

